# IL-22 promotes mucin-type O-glycosylation and MATH1^+^ cell-mediated amelioration of intestinal inflammation

**DOI:** 10.1016/j.celrep.2024.114206

**Published:** 2024-05-11

**Authors:** Ankita Singh, Michael Beaupre, Cecilia Villegas-Novoa, Kiyoshi Shiomitsu, Stephen J. Gaudino, Suzanne Tawch, Ruhee Damle, Cody Kempen, Biswa Choudhury, Jeremy P. McAleer, Brian S. Sheridan, Paula Denoya, Richard S. Blumberg, Patrick Hearing, Nancy L. Allbritton, Pawan Kumar

**Affiliations:** 1 Department of Microbiology and Immunology, Renaissance School of Medicine, Stony Brook University, Stony Brook, NY 11794, USA; 2 Department of Bioengineering, University of Washington, Seattle, WA 98195, USA; 3 GlycoAnalytics Core, University of California, San Diego, La Jolla, CA 92093, USA; 4 Department of Pharmaceutical Sciences, Marshall University School of Pharmacy, Huntington, WV 25701, USA; 5 Division of Colon and Rectal Surgery, Department of Surgery, Stony Brook University Hospital, Stony Brook, NY 11794, USA; 6 Division of Gastroenterology, Hepatology and Endoscopy, Department of Medicine, Brigham and Women’s Hospital, Harvard Medical School, Boston, MA 02115, USA; 7 Lead contact

## Abstract

The interleukin (IL)-22 cytokine can be protective or inflammatory in the intestine. It is unclear if IL-22 receptor (IL-22Ra1)-mediated protection involves a specific type of intestinal epithelial cell (IEC). By using a range of IEC type-specific *Il22Ra1* conditional knockout mice and a dextran sulfate sodium (DSS) colitis model, we demonstrate that IL-22Ra1 signaling in MATH1^+^ cells (goblet and progenitor cells) is essential for maintaining the mucosal barrier and intestinal tissue regeneration. The IL-22Ra1 signaling in IECs promotes mucin core-2 O-glycan extension and induces beta-1,3-galactosyltransferase 5 (B3GALT5) expression in the colon. Adenovirus-mediated expression of *B3galt5* is sufficient to rescue *Il22Ra1*^IEC^ mice from DSS colitis. Additionally, we observe a reduction in the expression of *B3GALT5* and the Tn antigen, which indicates defective mucin O-glycan, in the colon tissue of patients with ulcerative colitis. Lastly, IL-22Ra1 signaling in MATH1^+^ progenitor cells promotes organoid regeneration after DSS injury. Our findings suggest that IL-22-dependent protective responses involve O-glycan modification, proliferation, and differentiation in MATH1^+^ progenitor cells.

## In brief

Singh et al.’s study shows that the IL-22Ra1-B3GALT5 axis is important in intestinal epithelial cells to protect from DSS colitis. The intestinal epithelial target of IL-22 is MATH1^+^ cells, which regulate *B3galt5* expression (mainly goblet cells) and induce epithelial regeneration (MATH1^+^ progenitor cells) to protect from the development of DSS colitis.

## Graphical Abstract

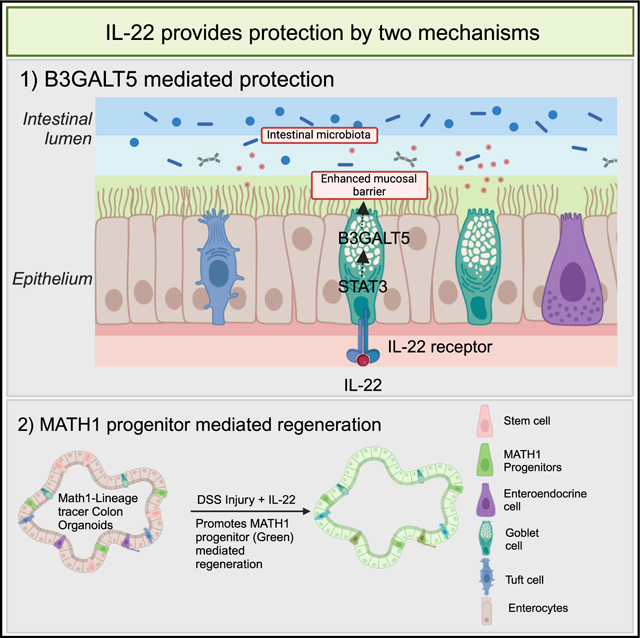

## INTRODUCTION

Inflammatory bowel disease (IBD), including Crohn’s disease and ulcerative colitis (UC), is characterized by disruptions in the protective lining of the intestine and an abnormal immune response.^1^ The importance of interleukin (IL)-22 in IBD is highlighted by the fact that genes associated with IBD (*IL-23R*, *IL-10R2*, and *STAT3*) are connected to the function of this cytokine.^2^ IL-22 plays a crucial role in maintaining the integrity of the intestinal epithelial barrier and regulating the immune response in the gut.^3^

The absence of IL-22 has been shown to exacerbate disease in dextran sulfate sodium (DSS)- or T cell-induced animal models of colitis.^4^ On the other hand, in certain animal models of colitis IL-22 has been shown to have a pro-inflammatory role.^5,6^ It should be noted that prolonged usage of IL-22 as a therapy may lead to the development of colitis-associated cancer.^7–9^ Thus, the effects exerted by IL-22 are complex, and more studies are needed to understand the functional dichotomy of the cytokine. It is also important to identify the major IL-22 responder cells and their effector proteins that induce protection since these sources can also be used as potential interventions to induce more specific effects.

Studies have shown that germline *Il22*^−/−^ and *Il22Ra1*^−/−^ mice are more susceptible to chemical- and pathogen-induced colitis.^4,10–14^ However, because IL-22Ra1 is expressed by various intestinal epithelial cells (IECs) such as secretory, absorptive, and intestinal stem cells (ISCs),^15^ it is unclear how IL-22 provides mucosal protection and what the specific role is of each cell type. Current tools do not allow for a precise understanding of the direct impact of IL-22Ra1 signaling on individual epithelial cell types. Previous research has demonstrated that IL-22 promotes epithelial cell regeneration through LGR5^+^ ISCs.^15^ However, recent studies suggest that IL-22 may also cause depletion of LGR5^+^ ISCs^16,17^ and that certain inflammatory responses can significantly decrease their numbers.^18^ Several studies have demonstrated the critical role of ATOH1/MATH1^+^ secretory progenitors in promoting IEC regeneration.^19,20^ It is unclear which type of IEC is targeted by IL-22 and whether it induces MATH1^+^ cell-mediated regeneration.

To investigate IEC type-specific IL-22 function, we generated *Il22Ra1*-specific entire gut epithelium (*Il22Ra1*^*IEC*^), tamoxifen-inducible *Lgr5-*^*Cre-ERT2*^ (*Il22Ra*^*ISC*^), Paneth (*Il22Ra1*^*Paneth*^), and RU-486-inducible MATH1 (*Il22Ra1*^*Math1-PGR*^) conditional knockout mice.

We subjected different cell-type-specific conditional knockout mice to DSS-induced intestinal inflammation. We observed that DSS administration resulted in a reduction in cell proliferation, beta-1,3-galactosyltransferase 5 (B3GALT5) expression, and Paneth cell number in *Il22Ra1*^*IEC*^ mice. Interestingly, our results showed that Paneth and LGR5^+^ cells were not necessary for IL-22-mediated protection against colon inflammation. B3GALT5 is an enzyme that plays a role in mucin-type O-glycan core extension.^21^ A recent study showed that the B3GALT5 enzyme protects from DSS colitis by promoting sialylation of MUC2.^22^ Our mass spectrometry results showed a defect in core-2 elongation of mucin-type O-glycan in naive *Il22Ra1*^*IEC*^ mice. We also found that adenovirus-mediated overexpression of the *B3galt5* gene helped to alleviate the weight loss and inflammatory phenotype of DSS-administered *Il22Ra1*^*IEC*^ mice. Our organoid data showed that IL-22 was directly responsible for the induction of the *B3galt5* gene. We observed that IL-22-mediated STAT3 activation was required to induce *B3galt5* expression. Since mainly goblet cells are responsible for producing mucus in the colon, we generated secretory cell-specific *Il22Ra1*^*Math1-PGR*^ mice. When subjected to DSS, *Il22Ra1*^*Math1-PGR*^ mice were more susceptible to intestinal inflammation. We observed significant increase in levels of the Tn antigen, which indicates less complex and less sialylated glycans in *Il22Ra1*^*Math1-PGR*^ mice. Additionally, we analyzed the glycosyltransferase defects in patients with UC and observed reduced *B3GALT5* expression in the colon of UC patients as compared to non-IBD controls. Our data also show that IL-22 induces *B3GALT5* expression in the human colon cell line. We found that the inflamed tissue of patients with UC had an increased expression of the Tn antigen. Lastly, using colon organoids of *Math1-PGR* lineage tracer mice, we show that IL-22Ra1 signaling in MATH1^+^ progenitor cells causes IEC regeneration. Collectively, our data show that the IL-22-dependent STAT3-B3GALT5 pathway and MATH1^+^ progenitor cell-mediated intestinal regeneration protected from DSS colitis.

## RESULTS

### Enhanced chemical-induced colitis susceptibility of *Il22Ra1*^*IEC*^ mice is independent of LGR5^+^ ISCs

**Figure 1.**
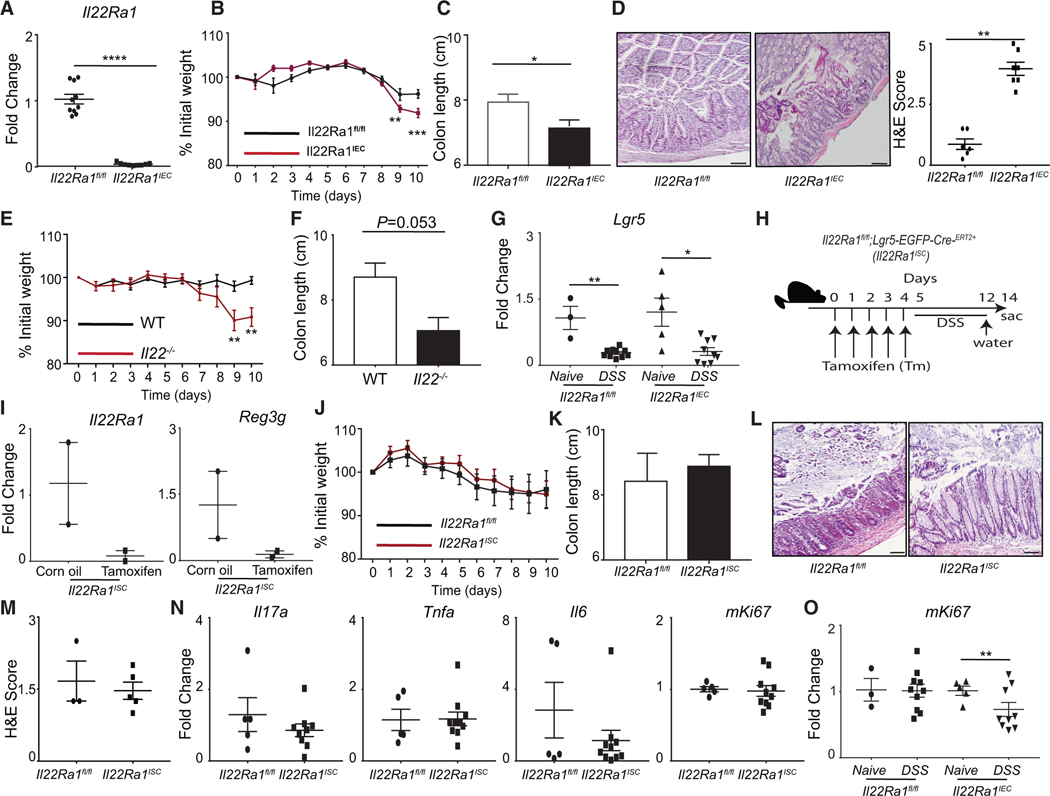
Enhanced susceptibility to DSS-induced colitis of intestinal epithelial cell-specific *Il22Ra1* knockout mice is independent of LGR5^+^ ISCs (A) RT-PCR shows *Il22Ra1* expression in the distal colon tissue of dextran sulfate sodium (DSS)-treated *Il22Ra1*^*fl/fl*^ and *Il22Ra1*^*IEC*^ mice. (B–D) Analysis of (B) weight loss, (C) colon length, and (D) H&E staining (50 μm, left) and histological score (right) in the distal colon tissue of *Il22Ra1*^*fl/fl*^ and *Il22Ra1*^*IEC*^ mice after DSS treatment. (E and F) Analysis of (E) weight loss with the start of DSS and (F) colon length in *Il22*^−/−^ and control (*C57BL/6J*) mice. (G) RT-PCR analysis of *Lgr5* expression in the distal colon tissue of naive and DSS-treated *Il22Ra1*^*fl/fl*^ and *Il22Ra1*^*IEC*^ mice. (H) Schematic representation of tamoxifen and DSS treatment of *Il22Ra1*^*ISC*^ and littermate control mice. (I) RT-PCR analysis for expression of *Il22Ra1* and *Reg3g* genes in the distal colon tissue of naive *Il22Ra1*^*ISC*^ mice with/without 5 days of tamoxifen injection. (J–N) Analysis of (J) weight loss, (K) colon length, (L) H&E staining (50 μm), (M) H&E scoring, and (N) RT-PCR of inflammatory cytokine genes (*Il17a*, *Tnfa*, and *Il6*) and *mKi67* expression in distal colon tissue of DSS-treated *Il22Ra1*^*fl/fl*^ and *Il22Ra1*^*ISC*^ mice. (O) RT-PCR analysis of *mKi67* expression in the distal colon tissue of naive and DSS-treated *Il22Ra1*^*fl/fl*^ and *Il22Ra1*^*IEC*^ mice. (A)–(C), (G), (J), (K), (N), and (O) are generated from at least 2 independent experiments. (D) and (L) are representative of at least 3 mice per group. Data are presented as mean ± SEM in the graphs. *p < 0.05, **p < 0.01, ***p < 0.001, and ****p < 0.0001 (two-way ANOVA or Mann-Whitney test, two-tailed).

To study the functional role of IL-22Ra1 in experimental colitis, we utilized IEC-specific *Il22Ra1* knockout mice (*Il22Ra1*^*IEC*^) as described.^14,23,24^ We validated reduced *Il22Ra1* and *Reg3g* expression in the distal colon of *Il22Ra1*^*IEC*^ mice (Figures 1A and S1A). Consistent with published work on germline *Il22*^−/−^ and *Il22Ra1*^−/−^ knockout mice,^4,12,14^ our data show significant weight loss, reduction in colon length, mucosal damage, and an increased expression of inflammatory cytokine genes in the distal colon of *Il22Ra1*^*IEC*^ mice (Figures 1B–1D and S1B). Since other cytokines such as IL-20 and IL-24 also signal through IL-22Ra1, we used *Il22*^−/−^ mice and observed that these mice were more susceptible to DSS-induced colitis as compared to wild-type controls (Figures 1E and 1F). Collectively, our results indicate that IL-22Ra1 signaling in IECs protects mice from DSS-induced development of colitis.

We observed increased expression of *Dclk1* (tuft cells) and *Chga* (enteroendocrine cells) in *Il22Ra1*^*IEC*^ as compared to *Il22Ra1*^*fl/fl*^ mice after DSS treatment (Figure S1C). Two studies have reported paradoxical findings on the role of IL-22Ra1 signaling in LGR5^+^ ISCs in intestinal epithelial regeneration.^15,16^ We observed a significant reduction in *Lgr5* expression in the distal colon of DSS-treated mice, regardless of Cre status (Figure 1G), which is consistent with a study showing the significant depletion of LGR5^+^ cells in the distal colon of mice after DSS treatment.^3^ The LGR5 depletion was not 100%, thus it is possible that they still contribute to the intestinal epithelial regeneration. To understand the role of IL-22Ra1 signaling in LGR5^+^ ISCs, we generated tamoxifen-inducible ISC-specific *Il22Ra1* knockout mice (*Il22Ra1*^*ISC*^) (Figure 1H). We validated knockout by analyzing the expression of *Il22Ra1* and *Reg3g* in the distal colon of tamoxifen-treated *Il22Ra1*^*ISC*^ mice (Figure 1I). We analyzed LGR5-GFP^+^ cells in the colon of naive mice and observed no difference in the number of crypts positive for LGR5-GFP^+^ cells in tamoxifen-treated as compared to corn-oil-treated *Il22Ra1*^*ISC*^ mice (Figure S1D). After tamoxifen and DSS treatment, an almost complete depletion of LGR5-GFP^+^ cells was observed in the distal colon of *Il22Ra1*^*ISC*^ mice (Figure S1D). This indicates that there was no significant reappearance of LGR5^+^ cells with intact IL-22Ra1 signaling when the mice were collected after treatment with DSS. We subjected *Il22Ra1*^*ISC*^ and their littermate control mice to DSS and found no difference in weight loss, colon length, histopathological changes, or inflammatory cytokine gene expression (Figures 1J–1N). In addition, there was no difference in cell proliferation gene (*mKi67*) expression in *Il22Ra1*^*ISC*^ and *Il22Ra1*^*fl/fl*^ mice (Figure 1N). However, a significant reduction in *mKi67* expression was observed in the distal colon of DSS-treated *Il22Ra1*^*IEC*^ mice (Figure 1O). Thus, IL-22Ra1 signaling in LGR5^+^ ISCs is dispensable in causing intestinal epithelial regeneration.

### The dispensable role of Paneth cell-specific (*Il22Ra1*^*Paneth*^) IL-22Ra1 signaling was observed in chemical-induced colitis

**Figure 2.**
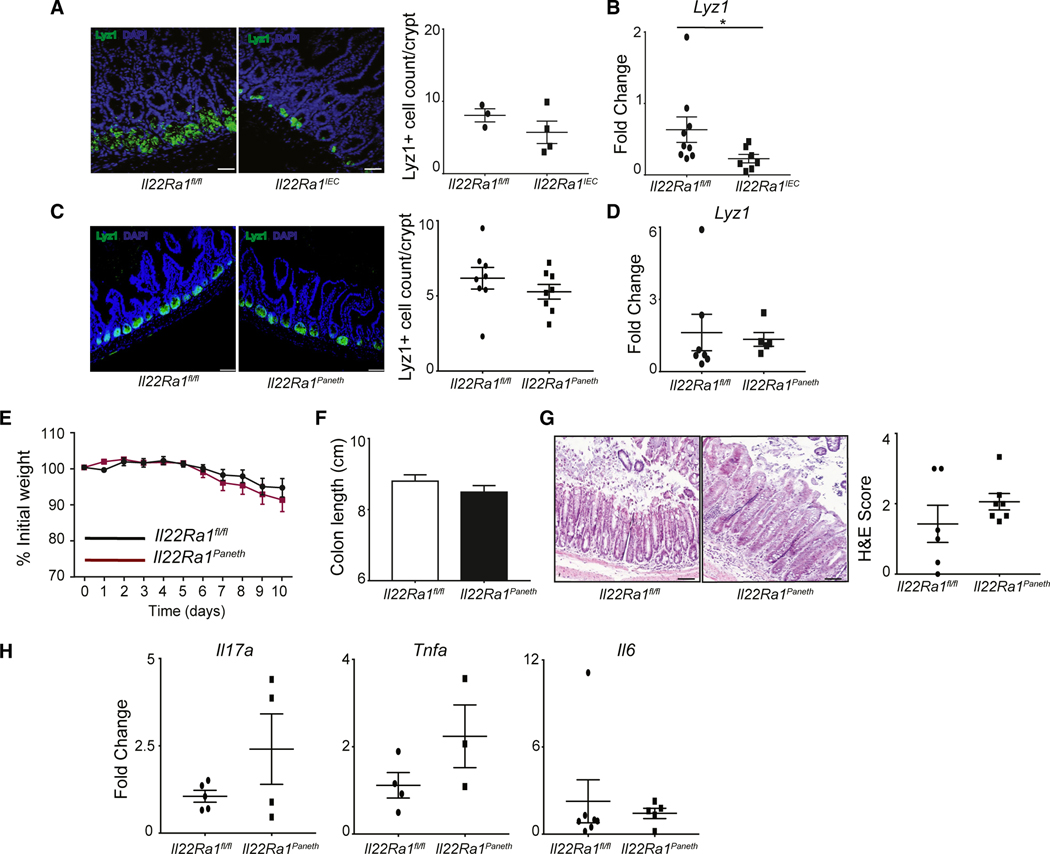
Dispensable role of Paneth cell-specific (*Il22Ra1*^*Paneth*^) IL-22Ra1 signaling in DSS-induced colitis (A and B) Representative image showing immunofluorescence LYZ1-stained cells (left, 50 μm) and their numbers (right) (A) and RT-PCR data for *Lyz1* expression (B) in the terminal ileum of DSS-treated *Il22Ra1*^*fl/fl*^ and *Il22Ra1*^*IEC*^ mice. (C and D) Representative image of immunofluorescence LYZ1-stained cells (left, 50 μm) and their numbers (right) (C) and RT-PCR analysis of *Lyz1* expression (D) in terminal ileum of DSS-treated *Il22Ra1*^*fl/fl*^ and *Il22Ra1*^*Paneth*^ mice. (E–H) Analysis of (E) weight loss, (F) colon length, (G) H&E staining (left, 50 μm) and scoring (right), and (H) RT-PCR for expression of inflammatory cytokine genes (*Il17a*, *Tnfa*, and *Il6*) in the distal colon tissue of naive and DSS-treated *Il22Ra1*^*fl/fl*^ and *Il22Ra1*^*Paneth*^ mice. (E) and (F) are generated from 3 independent experiments with at least 5 mice per group. (A), (C), and (G) are representative of at least 3 mice per group. Data are presented as mean ± SEM in the graphs. *p < 0.05 (two-way ANOVA or Mann-Whitney test, two-tailed).

Among IECs, Paneth cells have the highest expression of *Il22Ra1*.^24^ Also, IL-22Ra1 signaling in the Paneth cells induces antimicrobial peptide production, such as cryptidin, Reg3γ, and Reg3β.^24–28^ In addition, we and others have shown that IL-22 promotes Paneth cell differentiation.^24,29^ Paneth cells are absent in the mouse colon; however, their antimicrobial products (cryptidin, lysozyme, etc.) traverse to the colon lumen.^30,31^ It has been reported that Paneth-like cells have been found in the colon.^32^ It is not known if IL-22Ra1 signaling in the Paneth cells can affect the mucosal barrier in the colon and protect it from DSS-induced colitis development. After DSS treatment, we observed a modest reduction in LYZ1^*+*^ Paneth cell numbers and a significant reduction in *Lyz1* expression in the terminal ileum of *Il22Ra1*^*IEC*^ mice (Figures 2A and 2B). Thus, we used *Il22Ra1*^*Paneth*^ mice to explore if the lack of IL-22Ra1 signaling in Paneth or Paneth-like cells has an impact on coloninflammation. We have previously validated Paneth cell-specific *Il22Ra1* knockout mice.^24^ We observed no difference in the expression of inflammatory cytokine genes in the distal colon of naive *Il22Ra1*^*Paneth*^ and *Il22Ra1*^*fl/fl*^ mice (Figure S2A). We have previously shown a reduced number of Paneth cells in *Il22-Ra1*^*Paneth*^ mice at a steady state.^24^ However, after DSS treatment, we observed no difference in Paneth cell number or *Lyz1* expression in the terminal ileum of *Il22Ra1*^*Paneth*^ and *Il22Ra1*^*fl/fl*^ mice (Figures 2C and 2D). In addition, DSS-treated *Il22Ra1*^*Paneth*^ mice were equally susceptible to colitis, as indicated by no differences in weight loss, colon length, histopathological changes, and inflammatory cytokine gene expression (Figures 2E–2H). As DSS mainly causes damage in the colon, the frequency of Paneth-like (CD44^+^cKit^+^) cells in the colon has been analyzed.^32^ We observed no difference in the frequency of Paneth-like cells in the colon tissue of DSS-treated *Il22Ra1*^*Paneth*^ and *Il22Ra1*^*fl/fl*^ mice (Figure S2B).

Collectively, our data show that the lack of IL-22Ra1 signaling in the Paneth cell is not involved in exacerbating DSS-induced colitis.

### A defect in mucin-type O-glycosylation was observed in *Il22Ra1*^*IEC*^ mice

**Figure 3.**
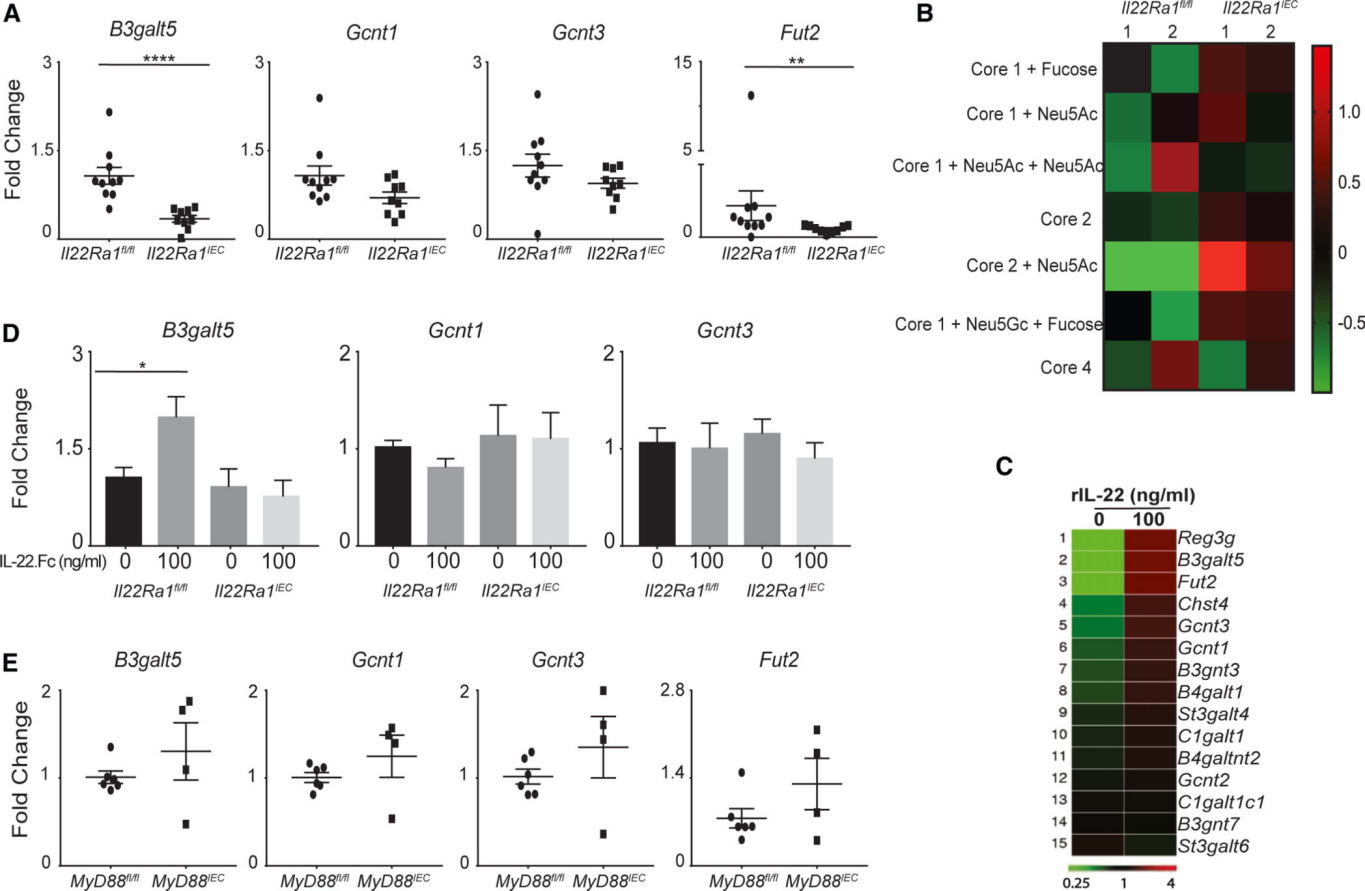
Defect in mucin-type O-glycan and glycosyltransferase expression in *Il22Ra1*^*IEC*^ mice (A) RT-PCR analysis of expression of various glycosyltransferase enzymes in the distal colon tissue of DSS-treated *Il22Ra1*^*fl/fl*^ and *Il22Ra1*^*IEC*^ mice. (B) Heatmap analysis showing the intensity of various O-glycan core structures (based on *m*/*z* ratio) in colon mucin by MALDI-TOF of naive *Il22Ra1*^*fl/fl*^ and *Il22Ra1*^*IEC*^ mice. (C) Heatmap analysis of RNA sequencing data of glycosyltransferase expression in small intestinal organoids of *C57BL/6J* mice treated with or without rIL-22 (100 ng/mL). (D) RT-PCR analysis of glycosyltransferase expression in colon organoids of *Il22Ra1*^*fl/fl*^ and *Il22Ra1*^*IEC*^ mice with/without IL-22.Fc (100 ng/mL). (E) RT-PCR analysis of glycosyltransferase expression in the distal colon tissue of DSS-treated *MyD88* ^*fl/fl*^ and *MyD88*^*IEC*^ mice. (A), (D), and (E) are generated from 2–3 independent experiments with at least 4 mice per group. Data are presented as mean ± SEM in the graphs. *p < 0.05, **p < 0.01, and ****p < 0.0001 (Mann-Whitney test, two tailed).

Our data show a significant reduction in *Muc1* and no difference in *Muc2* expression in the distal colon of DSS-treated *Il22-Ra1*^*IEC*^ mice (Figure S3A). It is to be noted that IL-22 is known to promote glycosylation in IECs.^14,33^ However, it is not known if IL-22 promotes type O-glycosylation of MUC2 (mucosal barrier forming mucin). Type O-glycosylation of mucin is required for protein stability and function and is mediated by various glycosyltransferase enzymes in goblet cells. IL-22 may alter the MUC2 type O-glycan profile rather than the protein expression. Indeed, we observed a significant reduction in the expression of glycosyltransferases such as *B3galt5* and fucosyltransferase 2 (*Fut2*) in the distal colon of DSS-treated *Il22Ra1*^*IEC*^ mice (Figure 3A). We analyzed the O-glycan profile of colon mucin of naive *Il22Ra1*^*IEC*^ and *Il22Ra1*^*fl/fl*^ mice by MALDI-TOF. We found that the lack of IL-22Ra1 signaling promoted a marked increase in premature termination of the core-2 structure (with sialic acid) and a modest increase in the core-2 structure in *Il22-Ra1*^*IEC*^ mice (Figure 3B). The increase in the formation of the core-2 structure is likely a feedback mechanism of the immature termination of glycan.

Previous studies have shown that the expression of glycosyltransferase is affected by gut microbes.^34,35^ It is well established that the lack of IL-22Ra1 signaling in IECs causes gut bacterial dysbiosis.^24^ We observed no difference in the expression of *B3galt5*, *Gcnt3*, and *Fut2* genes in the distal colon of germ-free as compared to *C57BL/6J* mice (Figure S3B). However, in germ-free mice, we observed a significant increase in the expression of the *Gcnt1* gene (Figure S3B). Next, we investigated if IL-22 directly regulates glycosyltransferase expression. Transcriptome analysis of IL-22-treated small intestinal organoids of *C57BL/6J* mice shows a significant increase in the expression of the following glycosyltransferases, *B3galt5* (489-fold), *Fut2* (74-fold), and *Gcnt3* (3.5-fold) (Figure 3C; GEO: GSE155172).^36^ A published study also reported a significant reduction in the expression of *B3galt5* in the ileum of *Il22*^−/−^ mice.^37^ We further show that upon IL-22 stimulation, a significant increase in *B3galt5* expression was observed in colon organoids from *Il22Ra1*^*fl/fl*^, while no change was observed in *Il22Ra1*^*IEC*^ mice (Figure 3D). Thus, IL-22Ra1 signaling in IECs, independent of gut microbiota, can induce *B3galt5* expression.

The basic mechanism and signaling pathways involved in the regulation of IEC glycosylation by gut microbes are unknown. We next explored if changes in *B3galt5* expression may be induced via Toll-like receptor (TLR) activation in the IECs. We induced colitis in mice lacking TLR signaling in IECs (*MyD88*^*IEC*^). After DSS treatment, *MyD88*^*IEC*^ mice developed severe colitis (Figures S3C and S3D). However, we did not observe any difference in the expression of *B3galt5* and *Fut2* genes in the distal colon of DSS-treated *MyD88*^*IEC*^ mice (Figure 3E). Collectively, our data show that IL-22-mediated regulation of *B3galt5* expression is independent of gut-microbiota-mediated TLR signaling in IECs.

### *B3galt5* overexpression protects *Il22Ra1*^*IEC*^ mice from DSS-induced colitis

**Figure 4.**
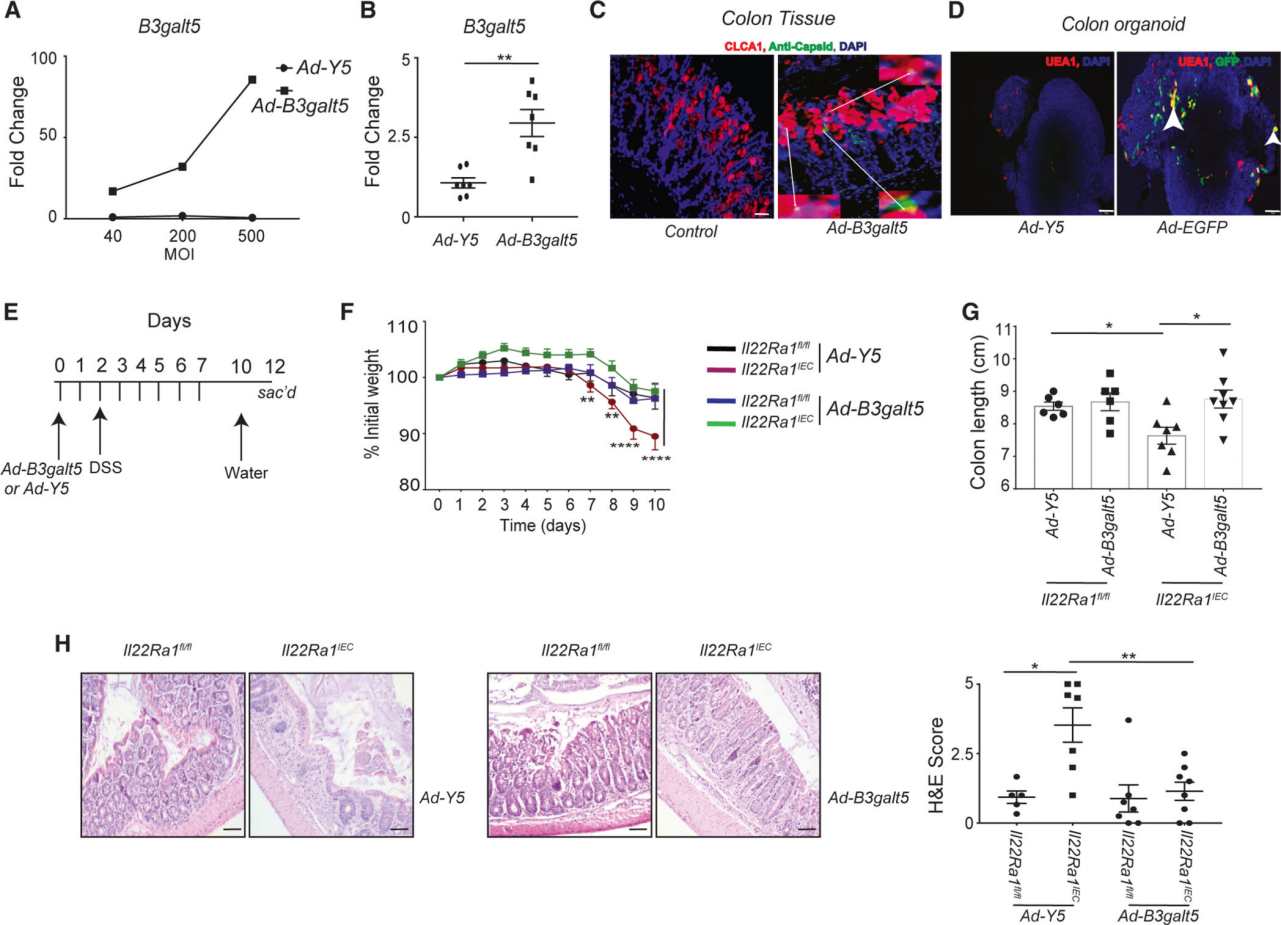
Administration of *Ad-B3galt5* vector protects *Il22Ra1*^*IEC*^ mice from DSS-induced colitis (A and B) RT-PCR analysis of *B3galt5* expression in (A) HEK-293 cell line at different multiplicities of infection and (B) *C57BL/6J* mice colon organoids transfected with either *Ad-B3galt5 or Ad-Y5* (control) vector. (C) Representative image of immunofluorescence adenovirus capsid (green), CLCA1 (red), and DAPI (blue) in colon tissue of *Il22Ra1*^*IEC*^ mice after 5 days of *Ad-B3galt5* i.p. injection (20 μm). (D) Representative image of immunofluorescence GFP (green), UEA I (red), and DAPI (blue)-stained colon organoids of *C57BL/6J* mice transfected with *Ad-EGFP* or *Ad-Y5* vector (50 μm). (E) Schematic representation of *Ad-B3galt5/Ad-Y5* and DSS treatment of *Il22Ra1*^*fl/fl*^ and *Il22Ra1*^*IEC*^ mice. (F–H) Analysis of (F) weight loss, (G) colon length, and (H) H&E staining (left, 50 μm) and scoring (right) of distal colon tissue of *Il22Ra1*^*fl/fl*^ and *Il22Ra1*^*IEC*^ mice after adenovirus and DSS treatment. (C) and (D) are representative of 1 independent experiment with at least 2 mice in each group. (F) is representative of 3 independent experiments with at least 5 mice in each group. Data are presented as mean ± SEM in the graphs. *p < 0.05, **p < 0.01, and ****p < 0.0001 (two-way ANOVA or Mann-Whitney test, two-tailed).

To understand the significance of B3GALT5 in providing IL-22-mediated protection from DSS-induced colitis. We custom synthesized an adenovirus vector expressing *B3galt5* (*Ad*–*B3galt5*, Vector BioLabs) and transfected HEK293 cells with *Ad-B3galt5* at different multiplicities of infection (MOIs; ratio of the number of virus particles to the number of target cells). The administration of *Ad-B3galt5*, but not an empty/control vector (*Ad-Y5*), induced *B3galt5* expression with a positive correlation with the MOI (Figure 4A). We next confirmed the efficiency of *Ad-B3galt5* transfection in the colon epithelium including goblet cells. We cultured the primary colon organoid from *C57BL/6J* mice and transfected them with *Ad-B3galt5.* Our data show a significant increase in the expression of *B3galt5* in colon organoids transfected with *Ad-B3galt5* (Figure 4B). To demonstrate *in vivo* transfection of IECs, we intraperitoneally (i.p.) injected *Ad-B3galt5* in *Il22Ra1*^*IEC*^ mice, and 5 days post-injection, the colon was stained for anti-adenovirus capsid and anti-CLCA1 antibody. We observed transfection of mature goblet cells (CLCA1^+^, adenovirus capsid^+^) along with other IECs (Figure 4C). Furthermore, goblet cell (UEA-1^+^)-specific transfection with an *Ad-EGFP* vector was observed in the colon organoids of *C57BL/6J* mice (Figure 4D).

Next, we evaluated the protective role of the B3GALT5 enzyme *in vivo*. The *Il22Ra1*^*IEC*^ and *Il22Ra1*^*fl/fl*^ mice were i.p. injected with *Ad-B3galt5* or *Ad-Y5* vector before inducing colitis (Figure 4E). Treatment with *Ad-B3galt5*, but not the *Ad-Y5* vector, rescued *Il22Ra1*^*IEC*^ mice from developing colitis, as revealed by weight loss and colon length data (Figures 4F and 4G). Histopathological analysis of distal colon tissue of *Ad-B3galt5*-administered *Il22Ra1*^*IEC*^ mice shows less epithelial cell damage and immune cell infiltration (Figure 4H). The expression of inflammatory cytokine gene (*Il6* and *Il1b*) was significantly reduced after *Ad-B3galt5* transfection of *Il22Ra1*^*IEC*^ mice (Figure S4A). Thus, we concluded that *B3galt5* plays a significant role in facilitating IL-22-mediated protection from colitis development.

### IL-22Ra1 signaling in MATH1^+^ cells plays a significant role in protecting from DSS-induced colitis

**Figure 5.**
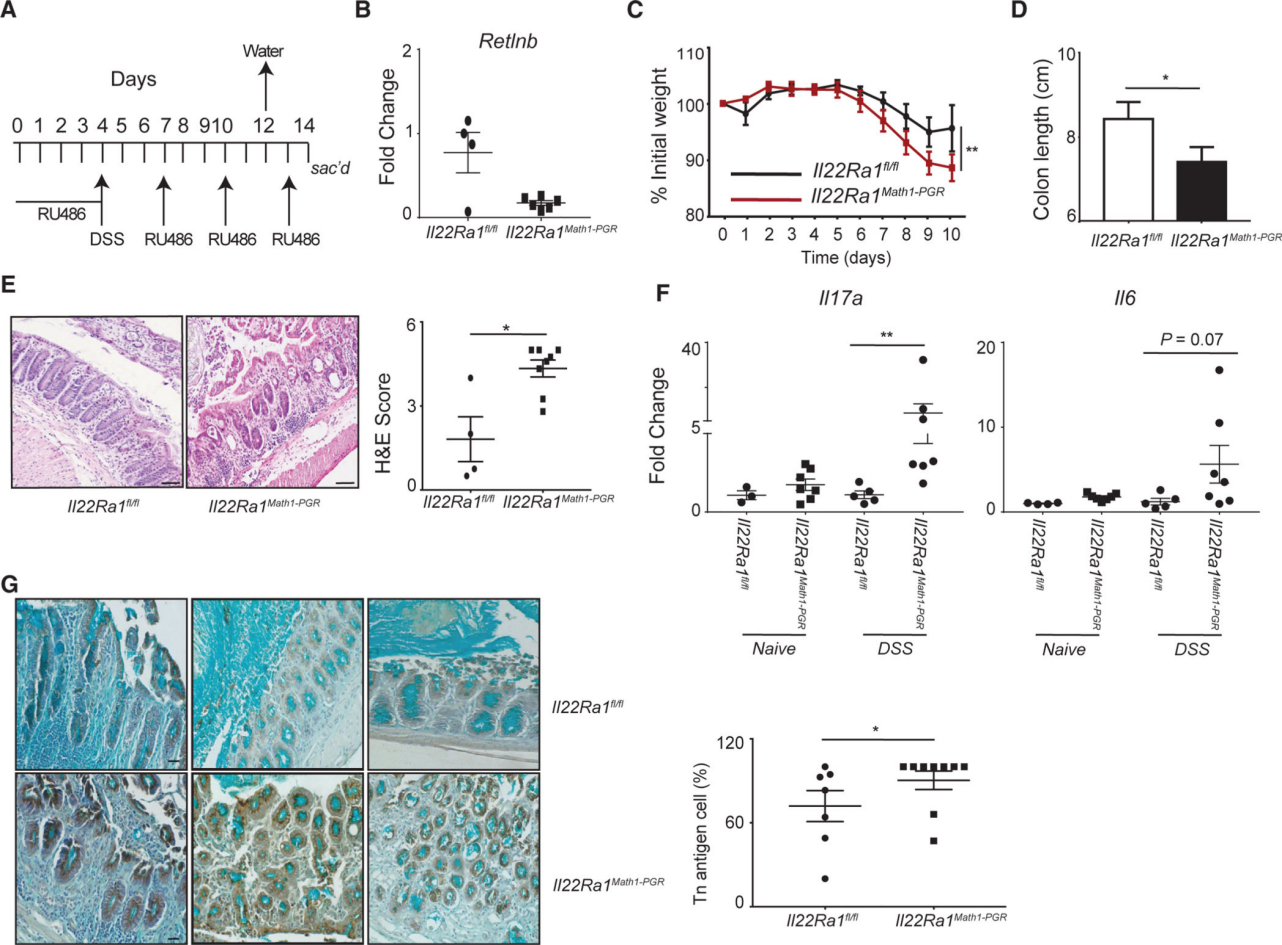
IL-22Ra1 signaling in MATH1^+^ cells plays a significant role in protecting from DSS colitis (A) Schematic representation of RU-486 and DSS treatment of *Il22Ra1*^*fl/fl*^ and *Il22Ra1*^*Math1-PGR*^ mice. (B–F) Analysis of (B) goblet cell-specific IL-22-inducible gene (*Retlnb*), (C) weight loss, (D) colon length, (E) H&E staining (left, 50 μm) and scoring (right), and (F) RT-PCR analysis of inflammatory cytokine genes (*Il17a*, *Il6*) in the distal colon tissue of DSS-treated *Il22Ra1*^*fl/fl*^ and *Il22Ra1*^*Math1-PGR*^ mice. (G) Representative image of immunohistochemically stained Tn antigen (brown) and Alcian blue (left panel) and their count (right) in the distal colon tissue of DSS-treated *Il22Ra1*^*fl/fl*^ and *Il22Ra1*^*Math1-PGR*^ mice (20 μm). (B)–(G) are representative of 4 independent experiments with at least 4 mice per group. Data are presented as mean ± SEM in the graphs. *p < 0.05 and **p < 0.01 (two-way ANOVA or Mann-Whitney test, two-tailed).

Goblet cells are the predominant producers of mucin in the colon. Since we observed reduced expression of the *B3galt5* gene that promotes mucin type-O glycosylation, we wanted to explore if IL-22Ra1 signaling in MATH1^+^ cells (predominantly goblet cells in the colon) induces *B3galt5* expression and protects mice from DSS-induced colitis. We generated RU-486-inducible MATH1^+^ cell-specific *Il22Ra1* knockout mice (Figure 5A). The knockout of *Il22Ra1* in MATH1^+^ cells was confirmed by analyzing goblet cell-specific IL-22-inducible *Retlnb* gene and IL-22Ra1 expression (Figures 5B and S5A). B3GALT5 is expressed by enterocytes and goblet cells.^22,38^ As expected, IL-22 stimulation of colon organoids of *Il22Ra1*^*Math1-PGR*^ mice resulted in *B3galt5* gene induction in both the control and *Il22Ra1* knockout groups. A significantly lower expression of *Retlnb* in response to IL-22 treatment in *Il22Ra1*^*Math1-PGR*^ mice confirmed a lack of goblet cell-specific response (Figure S6A).

We found significant weight loss, reduced colon length, and tissue damage in DSS-administered, RU-486-treated *Il22Ra1*^*Math1-PGR*^ mice (Figures 5C–5E). Additionally, a significant increase in expression of *Il17a* was observed in *Il22Ra1*^*Math1-PGR*^ mice (Figure 5F). However, we did not see a difference in the expression of glycosyltransferases, mucin, and other inflammatory cytokine genes (*Il1b*, *Tnfa*, and *Il6*) in the distal colon of naive or DSS-treated *Il22Ra1*^*Math1-PGR*^ mice (Figures S6B–S6D). We assume that the lack of a difference in *B3galt5* expression in *Il22Ra1*^*Math1-PGR*^ mice may be related to the enterocyte-specific IL-22 response.^22^ Collectively, our data show that IL-22Ra1 signaling in MATH1^+^ cells plays an important role in protecting from DSS colitis.

### Lack of IL-22Ra1 signaling in MATH1^+^ cells resulted in exposure of mucin core structure (Tn antigen)

A defective mucin-type O-glycosylation results in the exposure of a core base structure, termed as the Tn antigen. The sialylation of the Tn antigen on MUC2 protects it from proteolytic degradation by gut bacteria.^39^ The addition of sialic acid to the Tn antigen is carried out by the ST6GALNAC6 enzyme, which is dependent on the B3GALT5 enzyme. Indeed, sialylation of the O-glycan of mucin is reduced in *B3galt5*^−/−^ mice.^22^ Thus, a lack of the B3GALT5 enzyme (elongates O-glycan core structure and promotes sialylation) may result in the elevated expression of Tn antigen. As expected, we observed increased expression of the Tn antigen in *Il22Ra1*^*Math1-PGR*^ mice after DSS treatment (Figure 5G). Collectively, our data show that IL-22Ra1 signaling in MATH1^+^ cells may be important in protecting from DSS colitis by promoting the extension of the O-glycan structure and sialylation.

### Patients with IBD display dysregulated glycosyltransferase expression and IL-22 treatment induces *B3GALT5* expression in human colon cell line

To test the translational implication of our findings in human tissue, we explored the role of IL-22Ra1 signaling on mucus production, mucus layer thickness (mainly composed of MUC2), mucin-type O-glycosylation, and expression of the truncated O-glycan structure (Tn antigen) in human IECs. We cultured a human colonic epithelial cell monolayer to determine the effect of IL-22Ra1 signaling on goblet cell number (*MUC2* gene expression and MUC2 staining) and mucus layer thickness. We did not see a difference in MUC2 coverage on IL-22 stimulation of human intestinal monolayer cells during expansion and differentiation (medium conditions are different) as compared to blank medium (Figure S7A). However, *MUC2* expression reduced significantly once IECs were treated with IL-22 in the differentiation medium. This is similar to a published study that utilized organoid culture, wherein IL-22 stimulation resulted in a significant reduction of *Muc2*.^40^ Further, we used an air-liquid interface (ALI) culture technique to measure mucus thickness on IL-22 stimulation. IL-22 treatment of human IECs in ALI culture induced rapid proliferation and increased cell death when added to differentiation and expansion media or only to differentiation medium. We also observed a reduction in mucus layer thickness due to cell death (Figure S7B).

**Figure 6.**
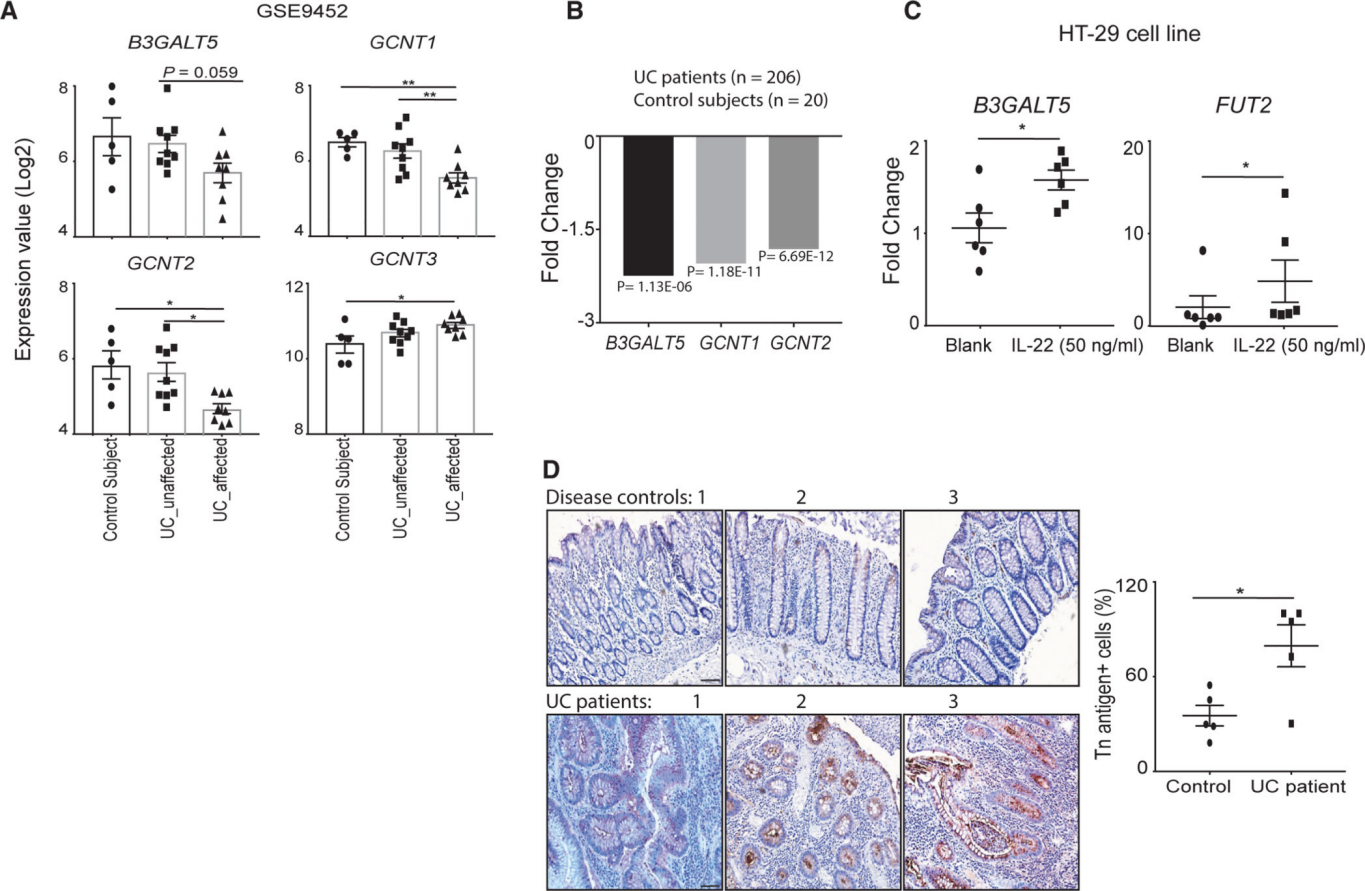
IL-22 induces *B3GALT5* expression in the human colon cell line and patients with UC express immature type O-glycan in colon tissue (A) Transcriptome analysis (GEO: GSE9452) for expression of glycosyltransferase genes in colon tissue of controls (*n* = 7), unaffected (non-inflamed) tissue (*n* = 9), or affected (inflamed) tissue (*n* = 8) of patients with UC. (B) Transcriptome analysis for expression of glycosyltransferase genes in rectal tissue of controls (*n* = 20) and patients with UC (*n* = 206). (C) RT-PCR analysis of *B3galt5* and *Fut2* genes upon stimulation of HT-29 cell line with human rIL-22 (50 ng/mL). (D) Representative images (3 individual patients) of immunohistochemically stained Tn antigen (brown) and nuclei stained with hematoxylin (left) and their percentage (right) in the colon tissue of diverticulitis (control, unaffected region) and patients with UC (affected region) (50 μm). (C) is representative of 2 independent experiments in triplicate. (D) comprise 5 tissue samples in each group. Data are presented as mean ± SEM in the graphs. *p < 0.05 and **p < 0.01 (Mann-Whitney test, two-tailed, Student’s t test in D).

We next analyzed the defect in the O-glycan synthesis pathway in human IBD studies. We analyzed UC patient colon tissue RNA sequencing data deposited at the GEO platform for the expression of various glycosyltransferases (GEO: GSE9452).^41^ We observed a trend in reduced expression of *B3GALT5* in affected as compared to unaffected colon tissue of patients with UC (Figure 6A). A significant reduction in *GCNT1* and *GCNT2* was observed in affected as compared to unaffected regions of colon tissue of patients with UC or healthy subject colon tissue (Figure 6A). The expression of *GCNT3* was significantly higher in affected colon tissue of patients with UC than in healthy controls (Figure 6A). Human studies consist of a highly heterogeneous population; due to the small sample size in the study, the difference in *B3GALT5* expression was not statistically significant. Indeed, another study, comprising a large sample size (*n* = 206) and a less heterogeneous UC patient population, shows significant downregulation in the expression of the *B3GALT5* gene (2.25-fold, Figure 6B) along with other glycosyltransferase genes as compared to non-IBD controls.^42^ We next show that IL-22 induces *B3GALT5* expression in human IECs by using the HT-29 cell line (colorectal adenocarcinoma). Upon IL-22 stimulation, a significant induction in the expression of *B3GALT5* and positive control *FUT2* was observed (Figure 6C).

Next, we show a significant increase in Tn antigen exposure in affected colon tissue of patients with UC as compared to the control sample (non-affected colon region of the patient with diverticulitis) (Figure 6D). An increased expression of immature glycan (Tn antigen) on mucin is reported in patients with active UC.^43–45^ Thus, our data suggest that B3GALT5-targeted therapy in a subset of patients with IBD may alleviate disease symptoms.

### Role of IL-22/STAT3 axis in inducing *B3galt5* expression

**Figure 7.**
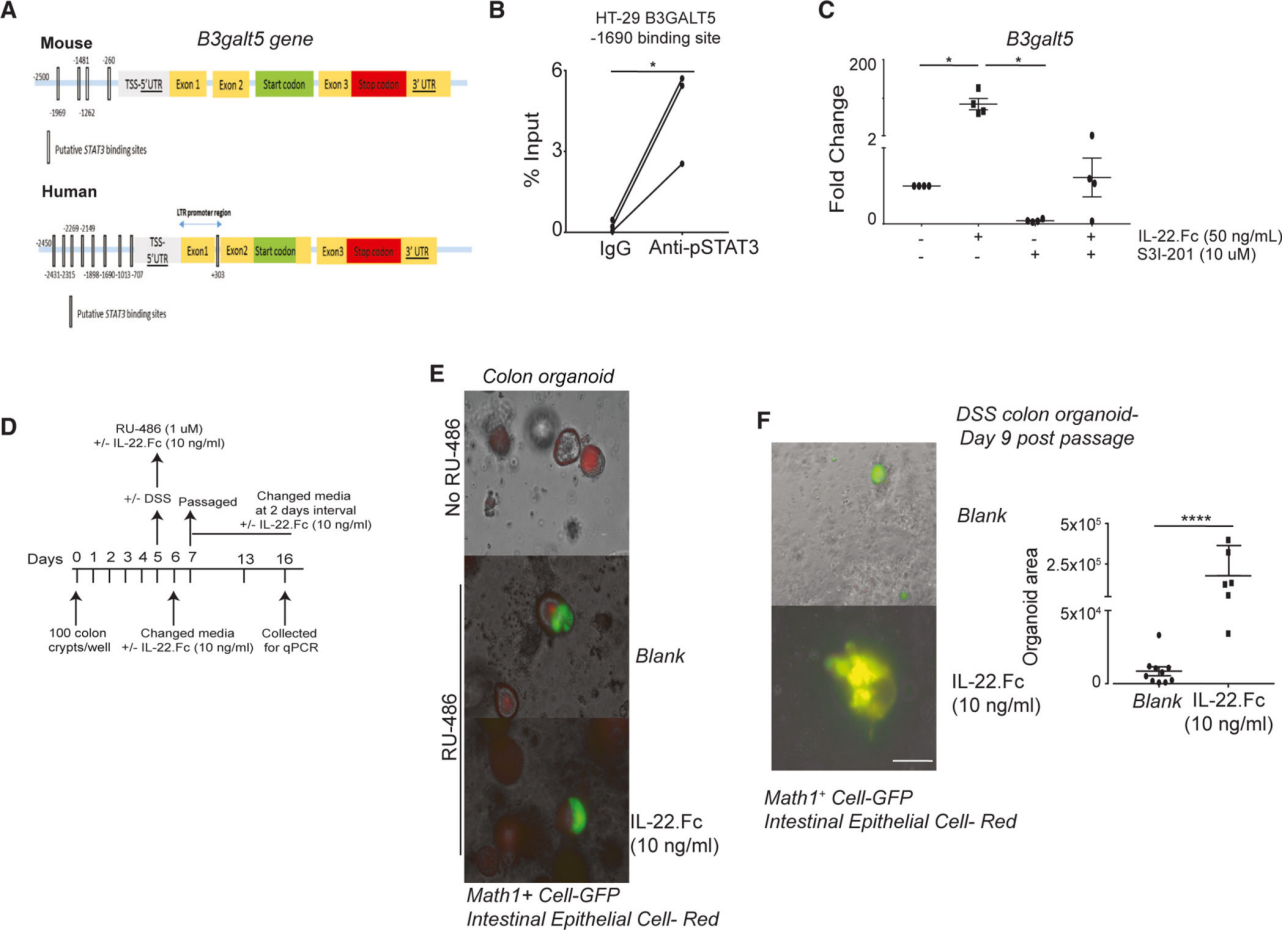
IL-22-STAT3 axis induces *B3galt5* expression and IL-22Ra1 signaling in MATH1^+^ progenitor cell induces intestinal epithelial regeneration after inflammation (A) Putative STAT3 binding site analysis by JASPAR in the promoter region of mouse and human *B3galt5* gene. (B) Chromatin immunoprecipitation assay in human rIL-22 (50 ng/mL, 24 h)-treated HT-29 cells with anti-pSTAT3 antibody. The pSTAT3-binding DNA was utilized for qPCR to determine the pSTAT3 binding site in the promoter region of the *B3GALT5* gene. (C) RT-PCR analysis of *B3galt5* gene expression in *C57BL/6J* mice colon organoids treated with/without mouse IL-22.Fc (50 ng/mL) and/or S3I-201 (STAT3 inhibitor, 10 μM). (D) Schematic representation of colon organoid treatment of *Math1-PGR* lineage tracer mice. (E) Image of IL-22.Fc- (10 ng/mL, 48 h) and RU-486-treated colon organoids of *Math1-PGR* lineage tracer mice (150 μm). (F) DSS ± IL-22.Fc (10 ng/mL)-treated colon organoid of *Math1-PGR* lineage tracer mice on day 9 post-passage with the organoid area on the right (150 μm). Green organoids represent MATH1^+^ progenitor cell-mediated regeneration after DSS treatment. (B) and (C) are representative of 2 independent experiments. Data are presented as mean ± SEM in the graphs. *p < 0.05 (Mann-Whitney test, two-tailed, paired Student’s t test in B). (E) and (F) are representative of 2 independent experiments with 5 mice in each group. Data are presented as mean ± SEM. ****p < 0.0001 (Mann-Whitney test, two-tailed).

Next, we investigated the involvement of the IL-22/STAT3 axis in *B3galt5* gene expression. We have analyzed mice and human *B3galt5* gene promoter regions for putative STAT3 binding sites. We identified a putative STAT3 binding site within 2,500 bp upstream of the transcription start site in the mouse and human *B3galt5* gene (Figure 7A). We stimulated the HT-29 cell line with IL-22 (50 ng/mL), and out of 9 putative binding sites, we identified the pSTAT3 binding site at —1,690 bp in the promoter region of the *B3GALT5* gene by chromatin immunoprecipitation-qPCR (Figures 7B and S8). Further, we treated colon organoids of *C57BL/6J* mice with STAT3 inhibitor and IL-22 or IL-22 alone. We observed an 85-fold reduction in the expression of the *B3galt5* gene with treatment by the STAT3 inhibitor and IL-22 as compared to treatment with IL-22 alone (Figure 7C). Thus, our data show that IL-22 via STAT3 activation induces *B3galt5* expression to protect from DSS-mediated colon injury.

### IL-22Ra1 signaling in MATH1^+^ progenitor cells promotes intestinal epithelial regeneration after inflammation

As we observed that *Il22Ra1*^*Math1-PGR*^ mice are more susceptible to DSS-induced colitis, we explored the additional IEC target of IL-22 to induce regeneration in response to inflammation. We and others show that treatment with DSS causes a significant loss of ISCs (LGR5^+^) in the mice colon (Figure 1G).^18^ The treatment of organoids with DSS results in a reduction of *Lgr5* expression and damage to the organoids (Figures S9A and S9B). Recent studies highlight the importance of ATOH1/MATH1^+^ cells in IEC regeneration after DSS-induced inflammation.^20^ We generated *Math1-PGR* lineage tracer mice to study the effect of IL-22Ra1 signaling in MATH1^+^ cells in mediating intestinal epithelial regeneration after DSS treatment. The expression of tdTomato is ubiquitous (red fluorescent protein), and GFP expression is induced in response to Cre recombination. Therefore, after RU-486 treatment, the mature secretory epithelium and MATH1^+^ progenitor cells will express GFP. Any cell type derived from MATH1^+^ progenitor cells will also express GFP. On day 5 of culture, the colon organoids of *Math1-PGR* lineage tracer mice were treated with RU-486 for 24 h along with IL-22 (Figure 7D). After 48 h of IL-22 treatment, we did not observe any difference in GFP expression or formation of whole organoids from GFP+ cells (green organoid). Thus, IL-22 treatment did not induce the proliferation or differentiation of MATH1^+^ cells in the absence of inflammation (Figure 7E). A previous study also reported that in the absence of inflammation, very few MATH1^+^ cells attain the stem cell-like properties.^20^ We next treated the organoids with DSS and observed an increased number of GFP^+^ (MATH1^+^) cells irrespective of IL-22 treatment. Interestingly, we found that continuous IL-22 treatment resulted in a significant increase in the size of GFP^+^ organoids on day 9 post-passage as compared to the control group (Figure 7F). Therefore, IL-22Ra1 signaling in MATH1^+^ cells causes intestinal epithelial regeneration after DSS-mediated depletion of LGR5^+^ ISCs.

## DISCUSSION

By using genetic mice models, we identified two pathways by which IL-22 protects from colitis development. One of the mechanisms is by mucin-type O-glycan chain elongation and sialylation, and the other relies on IL-22Ra1 signaling in MATH1^+^ progenitor cells to induce IEC regeneration after DSS-mediated injury.

The IL-22 cytokine is critical for maintaining homeostasis in the intestinal epithelium. This cytokine regulates multiple aspects of intestinal barrier functions, broadly categorized as epithelial regeneration, mucus secretion, and AMP production. Our study extensively explores the IEC target of IL-22 to mediate the beneficial effects of IL-22 in colon by using ISC-, Paneth cell-, and MATH1^+^ cell-specific *Il22Ra1* knockout mice models.

In the tamoxifen-inducible *Il22Ra1*^*ISC*^ mice model, it is likely that self-renewing ISCs may start to replenish the expression of IL-22Ra1 after 10 days of tamoxifen injection. However, after 8 days of DSS and 2 days of water treatment, we observed an almost complete depletion of LGR5-GFP^+^ cells in the colon, which eliminates the possibility of replenishment of IL-22Ra1 expression on self-renewing LGR5^+^ ISCs. Even after the depletion of LGR5^+^ ISCs, we did not observe any difference in *Ki67* expression in *Il22Ra1*^*ISC*^ as compared to *Il22Ra1*^*fl/fl*^ mice (Figure 1N). However, we observed a difference in *Ki67* expression in *Il22Ra1*^*IEC*^ mice, which indicates that after LGR5^+^ ISC depletion, IL-22 acts on other IECs or progenitors to induce the proliferation and regeneration of the intestine.

Mucin-type O-glycosylation steps involve the addition of sugar molecules to the core structure by several glycosyltransferases, which is independent of goblet cell differentiation program. We did not observe any defect in goblet cell differentiation; however, a significant reduction in glycosyltransferase (*B3galt5* and *Fut2*) expression was observed in the distal colon of DSS-treated *Il22Ra1*^*IEC*^ mice. The major class of glycan in gut mucin is type O-glycan, which constitutes around 80% of the mass of mucin, with *MUC2* being the most abundant mucin.^46^ Defects in glycosyltransferase enzyme expression will result in an immature/truncated type O-glycan profile of mucin in the colon. Any alteration in the glycan profile of mucin may result in compromised mucosal barrier function and pathological host and microbial interactions contributing to the development of colitis.^43,45,47–49^ A study shows that B3GALT5 promotes α2–6 sialylation of GlcNAc type-O glycan on MUC2 (disialyl Lewis A glycans) and limits gut bacterial interaction with IECs.^22^ The lack of the B3GALT5 enzyme resulted in a significant decrease in the thickness of the mucosal layer, increased bacterial dissemination in mesenteric lymph nodes, gut dysbiosis, and increased inflammatory response.^22^ B3GALT5 may also promote the elongation of the core-2 O-glycan structure of mucin along with sialylation of GlcNAc. The increased expression of truncated/immature glycans such as the Tn antigen on MUC2 protein is observed in patients with IBD.^43–45^ In line with these findings, we observed increased Tn antigen expression in the colon tissue of patients with UC and *Il22Ra1*^*Math1-PGR*^ mice, which may also indicate less sialylation (α2–6) of the Tn antigen.^22^ Thus, it is likely that B3GALT5 elongates the core-2 structure and promotes α2–6 sialylation.

The role of IL-22Ra1 signaling in promoting the fucosylation of IECs is shown in previous studies. A study shows that IL-22Ra1 signaling regulates the expression of *FUT2* and *B3GNT7*, which in turn promote fucosylation and affect host-microbe interactions.^14,50–52^ Another study shows that during inflammation-mediated gut dysbiosis, IL-22 is known to protect against the colonization of pathogens.^14^ The intestinal resistance against the colonization of pathogens did not depend on REG3γ but on IL-22-mediated changes in glycosylation. Although the study shows a significant reduction in *Fut2* expression, oral administration of 2′-fucosyllactose did not result in completely rescuing the mice from developing colitis,^14^ therefore indicating that other glycosyltransferase enzymes such as B3GALT5 may be involved, playing a significant role in providing protection. Using the *Ad-B3galt5* approach, we show that the *Il22Ra1*^*IEC*^ mice were completely rescued from developing DSS-induced colitis. However, the differences in rescuing mice from the development of colitis can also be attributed to the variation in animal models of colitis.

A study reported that the glycan profile of MUC2 is similar in normal controls and patients in remission, suggesting a role of inflammation in the regulation of mucin glycosylation.^45^ This suggests that *B3galt5* expression is driven by an inflammatory cytokine response. Similar to our finding, a previous study showed a significant induction in the expression of *B3galt5* after IL-22 stimulation of colon organoids from wild-type mice but not in *Il22Ra1*^−/−^ mice.^14^

The *B3galt5* expression is shown to be higher in germ-free mice as compared to conventional microbiota colonized mice.^34^ A study shows that TLR-signaling-mediated IL-22 production in dendritic cells regulates IEC fucosylation.^51^ However, we observed a direct effect of IL-22Ra1 signaling on *B3galt5* expression in primary organoid culture and observed no difference in glycosyltransferase expression in germ-free mice as compared to *C57BL/6J* mice. It is possible that the source (vendor-specific microbiota difference) of *C57BL/6J* (wild-type) mice may account for the difference in B3GALT5 expression. Our data on littermate *MyD88*^*IEC*^ mice show that *B3galt5* expression is independent of gut-microbiota-driven TLR signaling in IECs. However, lipopolysaccharide can prime dendritic or other innate immune cells by TLR activation to produce IL-22, which then acts on IECs to mediate mucosal healing.^11,53,54^ Therefore, gut microbes via innate immune cell activation and IL-22 production may regulate the expression of glycosyltransferase in IECs.

Further, we show the potential IEC target (MATH1^+^ cell) of IL-22 to mediate beneficial effects in the gut. In the colon, the MATH1^+^ cell population comprises mainly goblet cells (16%) and a smaller percentage of enteroendocrine (1%) and progenitor cell populations.^55,56^ Therefore, we generated *Il22Ra1*^*Math1-PGR*^ mice to study the role of IL-22Ra1 signaling in goblet cells. We show that *Il22Ra1*^*Math1-PGR*^ mice were more susceptible to DSS colitis. However, the lack of IL-22Ra1 signaling in MATH1^+^ cells did not result in any change in *B3galt5* expression as there are other types of IECs where IL-22Ra1 signaling is intact, such as enterocytes.^22^ The increased expression of the Tn antigen (truncated glycan and non-sialylated antigen) in *Il22Ra1*^*Math1-PGR*^ mice after DSS treatment further confirms the B3GALT5-associated defect in mucin-type O-glycan structure in colon. This indicates that IL-22Ra1 signaling in MATH1^+^ cells (goblet cells) plays an important role in regulating glycosyltransferase-mediated changes in the O-glycan structure of mucin and protecting from the development of colitis.

In addition, we observed that IL-22Ra1 signaling in MATH1^+^ progenitor cells plays a significant role in causing IEC regeneration after inflammation-induced depletion of LGR5^+^ ISCs. Although we did not observe any difference in the expression of *Ki67* in *Il22Ra1*^*Math1-PGR*^ mice after DSS treatment, the proliferation changes may likely be observed in the initial phase of DSS treatment when the mice start to lose weight.

Thus, our study shows that IL-22Ra1 signaling in IECs (and maybe goblet cells) plays an important role in regulating glycosyltransferase expression (*B3galt5*). Based on a study and our data, the B3GALT5 enzyme promotes elongation and sialylation of type-O glycan in MUC2 expressed by goblet cells.^22^ The elongation and sialylation of the O-glycan core structure results in the enhancement of gut barrier integrity and limit gut microbes’ interaction with IECs and their dissemination during inflammation.

### Limitations of the study

The limitation of the study is understanding the mechanism by which goblet cell-specific IL-22-STAT3-mediated *B3galt5* expression protects from colitis. Studying this further will help to identify the key mechanism that IL-22 uses to reduce the disease severity. Reduced *B3galt5* expression is associated with a truncated/immature O-glycan core structure and reduced disialyl Lewis A glycans, whereas increased exposure to the Tn antigen can also be associated with reduced sialylation. Further studies are needed to validate our findings in another model of experimental colitis and to explore the importance of IL-22Ra1 signaling in various types of IECs. Since we observed increased differentiation of IECs to tuft and enteroendocrine cells in *Il22-Ra1*^*IEC*^ mice after DSS treatment, it will be interesting to study the role of IL-22Ra1 signaling on these cells. We used colon organoids of Math1-PGR lineage tracer mice to study the role of IL-22 in causing MATH1^+^ progenitor cell-mediated epithelial regeneration. Additional *in vivo* studies using *Math1-PGR* lineage tracer mice are required to further support our findings.

## STAR★METHODS

### RESOURCE AVAILABILITY

#### Lead contact

Further information and requests for resources and reagents will be directed to and fulfilled by the Lead Contact, Pawan Kumar (pawan.kumar@stonybrook.edu).

#### Materials availability

This study did not generate new unique reagents.

#### Data and code availability

- The data reported in the study will be shared by the lead contact upon request.
- This paper does not report original code.
- Any additional information required to reanalyze the data reported in this paper is available from the lead contact upon request.

### EXPERIMENTAL MODEL AND STUDY PARTICIPANT DETAILS

#### Mice

All mice models used in our study were *C57BL/6J* backgrounds. The wild type (*C57BL/6J*), Villin Cre and B6.129 (Cg)-*Gt (ROSA) 26Sor*^*tm4(ACTB-tdTomato,-EGFP)Luo*^/J (mT/mG) mice were purchased from The Jackson Laboratory. *Il22Ra1* floxed (*Il22Ra1*^*fl/fl*^) mice characterization was performed as previously described.^23^ We received *Il22*^−/−^ from Dr. Jay K. Kolls (Tulane University), *Defa6-Cre* from Dr. Richard Blumberg (Harvard University), *MyD88* ^*fl/fl*^,*Villin-Cre* mice from Dr. Jeremy McAleer (Marshall University), *Math1-PGR-Cre* mice from Dr. Rodney D. Newberry (Washington University) and *Lgr5-EGFP-Cre*^*ERT2*^ mice from Dr. Vincent Yang (Stony Brook University, SBU). Germ-free *C57BL/6* mice tissues from Dr. Ajay Gulati (UNC Chapel Hill). Our study included both male and female mice aged 6–8 weeks. We received prior approval from the SBU Institutional Animal Care and Use Committee for all experiments involving animals.

#### Human tissue samples

The human tissue samples were obtained from biobank, a core facility of the department of Pathology and the Stony Brook Cancer Center. It archives and provides biological samples collected under informed consent. The clinical details of the patients with UC and diverticulitis is provided in Table S1.

#### DSS treatment

The mice were given drinking water containing either 2% or 2.5% DSS from MP Biologicals. We observed that the *Il22Ra1*^*IEC*^ and *Il22*^*Math1-PGR*^ mice were more vulnerable to intestinal injury caused by DSS. To induce colitis, these mice were provided with drinking water containing 2% DSS. Following mice models - *Il22Ra1*^*ISC*^, *Il22Ra1*^*Paneth*^ *(Defa6-Cre)*, and *MyD88*^*IEC*^ were given 2.5% of DSS in drinking water to induce colitis. We weighed the mice daily after administration of DSS. On day 8, we switched to normal water, and on day 10, we collected tissue and data after euthanizing the mice.

#### *Ad-B3galt5* injection

Adenovirus expressing *B3galt5* was custom synthesized at Vector BioLab. The *Il22Ra1*^*IEC*^ mice were injected i.p. with *Ad-B3galt5* or control vector *Ad-Y5* (10^9^ viral particles/mouse, Vector BioLabs) 2 days before the start of 2% DSS treatment.

#### Animal treatment

We i.p. injected tamoxifen (1 mg/mouse, Sigma) to *Il22Ra1*^*ISC*^ mice for 5 consecutive days. We collected the colon tissue to confirm the knockout of *Il22Ra1* in *Il22Ra1*^*ISC*^ mice. To induce colitis, we added 2.5% DSS (MP Biologicals) to drinking water on the last day of tamoxifen injection. For *Il22Ra1*^*Math1-PGR*^ mice, we injected RU-486 (200 μg/mouse, Sigma) i.p. for 5 consecutive days before DSS and then at an interval of 2 days. On the 5th day of RU-486 injection, we initiated 2% DSS water for the mice.

### METHOD DETAILS

#### RT-PCR

For RNA isolation and qPCR we followed a previously published protocol.^57^ In brief, we extracted RNA from the distal colon and terminal ileum tissue using the TRIzol method according to the manufacturer’s instructions (Life Technologies). Additionally, we followed the manufacturer’s guidelines (Qiagen Inc) to isolate RNA from organoids using the Qiagen RNeasy kit. Reverse transcription of RNA to cDNA was performed using a Biorad IScript kit (Thermo Fisher). For quantitative PCR, we used either 5 μL Biorad Sso advanced universal probes supermix or SYBR green supermix, along with 4.5 μL of cDNA and 0.5 μL of primer/probes. Relative quantification of gene expression was determined using *Hprt* or *Gapdh* as housekeeping genes. Primer details are given in the key resources table.

#### Lithium chloride purification

Lithium chloride (LiCl) purification was done before processing DSS-treated mice intestinal tissue for qPCR. The process involved incubating 25 μL RNA isolated from the tissue with 25 μL water, 30 μL of 8M LiCl, and 1 μL RNA-grade glycogen (20 mg) at −20°C for 1 h. We repeated this step with only LiCl after centrifuging the tube at 17000 RCF for 30 min. Next, we precipitated RNA with 10 μL of 3 M sodium acetate and 330 μL absolute ethanol overnight at −20°C. The following day, we pellet RNA and washed with 1 mL 70% ethanol, air dried, and resuspended in RNase-free water. Finally, we determined the concentration of RNA using a nanodrop.

#### Histology

Hematoxylin & Eosin (VWR) was done after sectioning the tissue as described.^57^ In brief, formalin-fixed and paraffin-embedded tissues were sectioned to 5 μm thickness using Leica microtome. The tissue sections were deparaffinized using Xylene and rehydrated in a descending gradient of ethanol (100%, 95%, 70%, water). Tissue was treated with hematoxylin for 30 s to stain the nuclei. The slides were washed for 15 min and then treated with Eosin for 15 min. The tissue was dehydrated using an ascending order of ethanol (70%, 95%, 100%) and Xylene. Finally, the slides were mounted with toluene and images were taken using a microscope (CKX41, Olympus). The scoring of tissue was done (blinded) on a scale of 0–6 as described in chemical induced colonic inflammation scheme 1.^58^

Tumor antigen (Tn, Human/mouse anti-Tn antibody, received from Dr. Cummings, Harvard Medical School) staining was done in colon tissue by immunohistochemistry assay. The tissue was rehydrated and boiled in a 10 mM citric acid buffer (pH 6, 10 min) and then treated with 0.3% of H2O2 (10 min). The tissue was washed with TBST (0.1% Triton X-100 in 1X Tris base buffer, pH 7.6) and blocked with 5% bovine serum albumin (BSA in 1X TBS) for 1 h at 37°C. The tissue was incubated with anti-Tn antigen antibody (5 μg/mL in TBST) overnight at 4°C. After washing, the tissue was incubated with horseradish peroxidase-labeled goat anti-human/mouse IgM antibody (1:400 dilution in TBST) for 1 h. The tissue was then incubated with 3,3′-Diaminobenzidine (Abcam) for 2 min at room temperature. Finally, the tissue was stained with hematoxylin or Alcian blue, and images were obtained using a microscope (CKX41, Olympus) after mounting the slide. The percentage of Tn^+^ cells was calculated by counting the crypt-villi and organized IEC structure areas positive for Tn antigen/total numbers of crypt-villi or organized IEC structures.

Immunohistochemistry: After rehydration and boiling of the tissue in a 10 mM citric acid buffer (pH 6, 30 min), we treated tissue with 0.3% of H_2_O_2_ (10 min). Blocking was done with 5% BSA in 1X TBS+0.1% Tween 20 for 1 h at 37°C and then incubated with biotinylated anti-GFP antibody (1:250 dilution, GeneTex) overnight at 4°C. The next day, we washed the tissue using 1X TBS+0.1% Tween 20 and added streptavidin-HRP (Abcam) conjugate for 30 min at room temperature. After washing, we incubated the tissue with DAB (Abcam) for 2–3 min and stained with hematoxylin (Sigma). The images were acquired after mounting the slides using a microscope (CKX41, Olympus).

Immunofluorescence staining: LYZ1 in the terminal ileum & adenovirus capsid and CLCA1 and UEA1 staining in colon tissue was done following a previously published protocol.^57^ The tissue was sectioned and rehydrated before being subjected to antigen retrieval by boiling with 10 mM citric acid buffer (pH 6) for 40 min. The tissue was permeabilized with PBST (1X PBS+ 1% BSA+ 0.1% Triton X-100) for 10 min at room temperature. To block the tissue, a solution of 5% BSA in 1X PBS was added and left for 1 h at 37°C. The tissue was then incubated overnight at 4°C with rabbit anti-human LYZ1-FITC (Dako, 1:50) or rabbit anti-mouse CLCA1 (abcam, 1:100) and anti-Capsid (1:100). The following day, the tissue was washed and respective secondary antibodies, goat anti-rabbit IgG-Alexa fluor 488 (Jackson ImmunoResearch, 1:200) or anti-rabbit IgG-Alexa fluor 647 (Jackson ImmunoResearch, 1:200) and goat anti-mouse IgG- Alexa Fluor 488 (Jackson ImmunoResearch, 1:200), was added for 1 h at 37°C. To visualize the nuclei, a DAPI hard stain (Vector Laboratories, H-1500) was used and the slide was mounted. Images were captured using a fluorescent microscope (CKX41, Olympus).

#### Cell lines

HEK293 cells were cultured in DMEM media supplemented with 10% fetal bovine serum (FBS, Gibco) and 1X penicillin-streptomycin-glutamine (Invitrogen). Plated 5×10^5^ cells/well and transfected with *Ad-B3galt5* or *Ad-Y5 vector* at various multiplicity of infection (MOI 40, 200, 500) in respective wells. Cultured cells for 2 days at 37°C and 5% CO_2_ and collected cells in 350 μL RLT (Qiagen micro RNA isolation kit) buffer with beta-mercaptoethanol (1:100 dilution, Sigma-Aldrich) for qPCR.

HT-29 cells were cultured in Macoy’s 5A high media (Gibco) supplemented with 10% FBS (Gibco) and 1X penicillin-streptomycin-glutamine (Gibco). HT-29 cells (1×10^5^ cells/well) were plated in 12 well plates (Corning) and stimulated with human rIL-22 (R&D, 50 ng/mL). Cells were collected after 24 h of culture in the RLT buffer for qPCR.

#### ChIP-qPCR

The NM-033172 transcript of Human *B3GALT5* gene with LTR-promoter was selected to design primers for ChIP-PCR assay. HT-29 cells (4×10^5^ cells/petri plate) were cultured for 24 h and then stimulated with human rIL-22 (50 ng/mL, R&D) for 24 h. The cells were harvested and processed for ChIP assay using a Simple ChIP Enzymatic Chromatin IP kit (Cell Signaling Technology, 9003S) as per the manufacturer’s instructions. Anti-pSTAT3 antibody (Abcam, 1:100) was used to pull down STAT3 binding DNA. There are 9 putative STAT3 binding sites in the promoter region of the *B3GALT5* gene. The target DNA fragments were quantified by qPCR with primers listed in the key resources table.

#### Organoid culture

For organoid culture, we used a published protocol.^57^ For organoid culture, the colon tissue (entire tissue) was flushed with cold 1X PBS and cut open longitudinally and then into 1 cm pieces. We collected the tissue in 40 mL of 1X PBS with 7.5 mM EDTA and incubated at 4°C on a shaker (60 rpm) for 30 min. We washed the tissue with PBS and gently shook in 30 mL PBS for 1 min before filtering it (70 μm, BD biosciences) in a conical tube containing 300 μL of fetal bovine serum (FBS). We repeated these steps two more times and centrifuged the three fractions of the supernatant at 350 RCF for 5 min. We pooled the pellet in 1 mL of PBS in a 1.5 mL tube and re-suspended it in matrigel (Corning, 100 crypts in 30 μL/well) before placing it in 24 well plates. After allowing the matrigel to polymerize at 37°C for 5 min, we overlayed 500 μL of growth medium (1:1 L-WRN and DMEM/F12) containing growth factors, 1X penicillin-streptomycin-glutamine (Invitrogen) and cultured the organoids at 37°C with 5% carbon dioxide, changing the medium after every 1 day. We added the following growth factors to L-WRN media for 2 days: 1X B27 (Invitrogen), 500 nM A83–01 (Tocris), Primosin, mEGF, 1X N2 (Invitrogen), 1mM N-acetylcysteine (Sigma-Aldrich), 10 mM Y-27632 (Sigma-Aldrich) and 10 mM CHIR 99021 (Tocris). We changed the media every 2 days but excluded CHIR and Y-27632.

On day 5, organoids were stimulated with 50/100 ng/mL of mouse IL-22.Fc (Evive Biotech, China) for 24 h. Colon organoids of *Il22*^*Math1-PGR*^ mice were treated with 800 ng/mL RU-486 24 h before IL-22.Fc (Evive Biotech, China) stimulation. Organoids were collected in the RLT buffer (Qiagen RNeasy Micro kit) for qPCR. For the *Ad-EGFP/Ad-B3galt5* transfection experiment, on day 3 of the colon organoid culture of *C57BL/6J* mice, 10^5^ viral particles of the *Ad-EGFP/Ad-B3galt5* vector were added to the media. After 2 days, *Ad-B3galt5* treated organoids were collected in the RLT buffer for qPCR and *Ad-EGFP* treated organoids were stained for GFP and UEA1 as described before.^57^

To stain the organoids, the organoid was collected in 500 μL of cell recovery medium (Corning) and incubated at 4°C for 30 min to depolymerize the gel. The organoids were then pelleted at 180 RCF for 5 min at 4°C and fixed with 4% paraformaldehyde (Electron Microscopy Sciences) for 30 min. After washing, the organoids were treated with PBST (1% BSA+0.1% Triton X-100) for 10 min and incubated with mouse anti-GFP antibody (Cell Signaling, 1:100) and mouse anti-UEA I Dylight 649 antibody (Vector Laboratories, 1:100) overnight at 4°C. The next day, the organoids were incubated with secondary goat anti-mouse IgG-Alexa fluor 488 (Jackson ImmunoResearch, 1:200) antibody for GFP staining for 1 h at 37°C. The organoids were transferred inside the IHC PAP pen circle onto a slide. To visualize the nuclei, a DAPI hard stain (Vector Laboratories, H-1500) was used to mount the slides. The images were acquired using a fluorescent microscope (CKX41, Olympus).

#### Flow cytometry

Followed the same protocol as described in the organoid culture experiment for crypt and epithelial cell isolation from colon tissue. The IECs were incubated with DMEM (10% FBS+ 1X Pen/Strep) for 20 min at 4°C. Then washed with PBS and single cell suspension was prepared by treating the cell with TrypLE (Invitrogen) for 5 min. Then added an equal volume of DMEM (10% FBS+ 1X Pen/Strep) and pipette 9–10 times to get a single cell suspension. We pellet down the cells and stained with anti-cKit-PE-Cy7 (1:100, Biolegend), anti-CD44-PerCp-Cy5.5 (1:100, Invitrogen), anti-CD45-APC-efluor-780 (1:100, Invitrogen), anti-EPCAM-efluor450 (1:100, Invitrogen), primary anti-CLCA1 (1:100, Abcam), anti-IL-22RA1-PE (1:20, R&D) and live/dead aqua (1:500, Invitrogen) dye for 30 min at 4°C. We fixed the cells with IC fixation buffer (Invitrogen) for 30 min at 4°C. Secondary anti-rabbit-Alexa fluor-488 antibody for CLCA1 staining (1:100, Jackson ImmunoResearch Labs) was added for 30 min at 4°C. The cells were washed and acquired using Cytek Aurora spectral flow cytometer (Cytek Biosciences).

#### STAT3 inhibitor treatment of organoids

On day 5 of culture, we treated *C57BL/6J* mice small intestinal organoids with S3I-201, a STAT3 inhibitor, at a concentration of 10 μM. After 30 min, we added mouse IL-22.Fc (Evive Biotech, China) at a concentration of 50 ng/mL with or without the STAT3 inhibitor. After 24 h of stimulation, we collected the organoids in the RLT buffer for qPCR analysis.

#### Colon mucin O-glycan profile

To collect mouse colon mucin, we cut the 3–5 cm colon tissue longitudinally and carefully removed the feces without disrupting the mucus layer. We used a clean glass slide to scrape the lumen (3–5 cm tissue) and collected mucin in 500 μL of water. To determine the O-glycan profile of mucin, we performed MALDI-TOF analysis at GlycoAnalytics core, University of California, San Diego, USA. We extracted O-glycans from the mucin samples using reductive beta elimination. To remove O-glycans, we treated the mucin samples with 0.5 M sodium hydroxide and 1 M sodium borohydride at 45°C for 16 h. We used Dowex resin to neutralize and remove excess sodium. The sample was re-evaporated using anhydrous methanol to remove boric acid and passed through a C18 cartridge. After lyophilizing the flow-through, we used MALDI-TOF/TOF mass spectrometry in positive reflectron mode in a Bruker Autoflex instrument. We analyzed the spectral data using GlycoWork Bench software. BSM-IS was included as a positive control, showing the presence of a mono and sialylated Core-1 structure having both Neu5Ac and Neu5Gc as non-reducing terminal sugar.

#### IL-22 effect on the number of goblet cells in submerged culture

Human colonic stem cells (male, 23 years old, RRID: 121 CVCL_ZR41) were expanded as described.^59^ Passaged primary cell to ≥80% confluency on day 3 following a published protocol.^59,60^ To investigate the impact of IL-22 on colon goblet cell differentiation and mucus secretion, human epithelial cells were grown on Transwells coated with 1% Matrigel in phosphate-buffered saline (PBS) at 37°C for 24 h. The cells were then transferred from 1 well (6-well plate) to 3 transwells (24-well plate) and expanded in expansion medium (EM) with or without human rIL-22 for 3 days, with daily medium changes (Table S2). On day 4, the medium on the luminal and basal side was changed to differentiation medium-submerged (DM-SUB) with or without human rIL-22 for differentiation (Table S2). On day 7, the cells were fixed with Prefer solution (Sigma) for 20 min, permeabilized with 0.5% Triton X-100 for 20 min, and blocked with 50 mM glycine at 20°C for 1 h. Anti-MUC2 antibodies (1:100; Santa Cruz Biotechnology) and Hoechst (1 μg/mL, ThermoFisher) were added to 200 μL of PBS and incubated with the cells at 4°C for 24 h. The cells were then washed and imaged for Hoechst and MUC2 using a confocal microscope (Olympus FluoView, Waltham, MA). Image analysis was performed using Fiji in ImageJ software (version 2.1.0/1.53c).

#### IL-22 effect on mucus thickness

Cells were transferred to transwells using EM medium (+/− IL-22) for 3 days. On day 4, the basal medium was changed to DM-air liquid interface (ALI) (+/− IL-22), and the luminal side medium was aspirated, creating an ALI culture.^61^ On day 7, cells were incubated for 1 h at 37°C with Hoechst. Red fluorescent beads 1 mg (10–45 μm diameter, Cospheric innovations in Microtechnology) were pipetted as a powder onto the mucus surface. The thickness of mucus was calculated by the distance between the red fluorescent beads on top of mucus and Hoechst-stained living epithelial cell nuclei by constructing z stack images using a confocal microscope and Cell Sens Dimension v1.18 software (Olympus Corp.). The nuclear fluorescence area was calculated using Fiji ImageJ software, and the nuclei number was counted using Imaris x64 v9.8.2.

#### *Math1-PGR* lineage tracer mice colon organoid treatment

Colon organoids were treated with 0.1% DSS for 3 h on day 5 of culture. Followed by DAPT (Notch inhibitor, 10 μg/mL) treatment for 3 h and then RU-486 (1 μM) for 24 h (10 ng/mL, +/− IL-22.Fc). We passaged organoids on day 3 (10 ng/mL, +/− IL-22.Fc) of DSS treatment and acquired images using an Evos fluorescence microscope on day 9 post passage (Thermo Fisher Scientific). The organoids size was determined using Fiji ImageJ software.

Lgr5-GFP-lineage tracer mice colon organoids were cultured for 5 days and then were treated with 0.1% DSS for 3 h followed by DAPT (Notch inhibitor, 10 μg/mL) treatment for 3 h. After 24 h of with/without DSS treatment, the organoids were imaged using Olympus CKX41 microscope and collected in an RLT buffer for analysis of *Lgr5* expression by qPCR.

GSE9452 transcriptome data: We analyzed the transcriptome data of GSE9452 using the NCBI GEO2R portal. The analysis involved creating three groups: UC affected tissue (*n* = 8), UC unaffected tissue (*n* = 9), and control tissue (*n* = 7) samples. To study specific genes, we used their identity numbers in the profile graph. After analyzing the samples, we downloaded readings for each gene to plot a graph.

#### STAT3 binding motif

To locate the promoter region of the mouse and human B3galt5 gene, NCBI Entrez was utilized. The eukaryotic promoter database provided the −2500 bp sequence from the transcription start site. JASPAR software was then employed to examine the promoter sequences for STAT3 binding sites.

### QUANTIFICATION AND STATISTICAL ANALYSIS

To determine statistical significance, two-way ANOVA, Mann-Whitney test, and Unpaired Student t test and Paired Student t test analysis were performed using GraphPad Prism 7 software (San Diego, California, USA). The data is presented as Mean ± Standard error of the mean (SEM) as shown in figure legends. The *p* value <0.05 was considered statistically significant. The significance levels are marked as *p < 0.05, **p < 0.01, ***p < 0.001, and ****p < 0.0001. The number of replicates for each of the experiments is mentioned in the figure legends.

## Supplementary Material

1

Supplementary Tables

## Figures and Tables

**Table:** KEY RESOURCES TABLE

REAGENT or RESOURCE	SOURCE	IDENTIFIER
Antibodies		

Rabbit anti-Human Lysozyme FITC	Dako	Cat#: F037201; RRID: AB_578661
Goat anti-rabbit IgG AF488	Jackson ImmunoResearch Labs	Cat#:111–545-144; RRID: AB 2338052
Mouse anti-GFP	Cell Signaling Technology	Cat#: 2955; RRID: AB 1196614
Goat anti-mouse IgG AF488	Jackson ImmunoResearch Labs	Cat#:115–546-003; RRID: AB 2338859
UEA-I Dylight 649	Vector Laboratories	Cat#: DL-1068
Mouse/Human anti-Tn	Dr. Richard D Cummings, Harvard Medical School	N/A
Mouse anti-GFP antibody (biotin)	GeneTex	Cat#: GTX26658; RRID: AB 371422
Mouse anti-IL22Ra1-PE	R&D	FAB42941P; RRID: AB 1964624
Mouse anti-CD45-APC-eFluor 780	Invitrogen	47–0451-82; RRID: AB 1548781
Mouse anti-EpCAM-eFluor-450	Invitrogen	48–5791-82; RRID: AB 10717090
Rabbit anti-mouse CLCA1	Abcam	Ab180851; RRID: AB 2722611
Recombinant Anti-STAT3 (Phospho Y705)	Abcam	ab76315; RRID: AB 1658549
Adenovirus hexon protein antibody (3G0)	Santa Cruz	Sc-80671; RRID: AB 1118724
Mouse PE/Cy7 anti-cKit	Biolegend	Cat#135111; RRID: AB 2131136
PerCP-Cy5.5 anti-mouse CD44	Invitrogen	45–0441-80; RRID: AB 925747
Live/Dead fixable Aqua dead cell stain kit	Invitrogen	L34957
IC fixation Buffer	Invitrogen	00–8222-49
Horseradish Peroxidase labeled Goat anti-human/mouse IgM	Southern Biotech	Cat#: 1020–05; RRID: AB 2794201

Chemicals, peptides, and recombinant proteins		

VECTASHIELD^®^ HardSet^™^ Antifade mounting medium with DAPI	Vector Laboratories	Cat#: H-1500
Mouse HRP/DAB ABC detection IHC kit	Abcam	Cat#: ab64264
SsoAdvanced^™^ Universal Probes Supermix	Bio-Rad	Cat#: 1725281
SsoAdvanced^™^ Universal SYBR^®^ Green Supermix	Bio-Rad	Cat#: 1725271
Simple ChIP Enzymatic Chromatin IP kit	Cell Signaling Technology	Cat#: 9003S
iScript^™^ Reverse Transcription Supermix	Bio-Rad	Cat#: 1708840
High-Capacity cDNA Reverse Transcription Kit	Thermo Fisher	Cat#: 4368814
Roche Diagnostics LIGHTCYCLER 480 SYBR GREEN	Roche	Cat#: 04887352001
EDTA	Invitrogen	Cat#: AM9260G
DSS	MP Biomedicals	Cat#: 160110
Alcian blue	Alfa Aesar	Cat#: J60122
Tamoxifen	Sigma-Aldrich	Cat#: T5648
DMSO	ThermoFisher	Cat#: BP231
DMEM/F12	Gibco	Cat#: 12634–010
PBS	Corning	Cat#: 21040CV
Fetal bovine serum	Gibco	Cat#: SH30071.03HI
Nuclear Fast Red	Electron Microscopy Sciences	Cat#: 26078–05
Hematoxylin	VWR	Cat#: 95057–844
Eosin	VWR	Cat#: 95057–848
Matrigel Matrix	Corning	Cat#: 356231
100x penicillin-streptomycin-glutamine	Gibco	Cat#: 10378016
Penicillin-Streptomycin solution, 100X	Corning	Cat#: 30–002-CI
L-Glutamine	Corning	Cat#: 25–005-CI
N2	Gibco	Cat#: 17502048
B27	Gibco	Cat#: 17504044
Mouse EGF	Peprotech	Cat#: 315–09
N-acetylcysteine	Sigma-Aldrich	Cat#: A9165
Gastrin-Leu15	Sigma-Aldrich	Cat#: G9145
A83–01	Tocris Bioscience	Cat#: 2939
Y-27632	Sigma-Aldrich	Cat#: Y05030
Cell Recovery Medium	Corning	Cat#: 354253
2-mercaptoethanol	Sigma-Aldrich	Cat#: M6250
Triton X-100	Sigma-Aldrich	Cat#: T8787
Tween 20	VWR	Cat#: 0777
CHIR99021	Tocris Bioscience	Cat#: 4423
Primocin	InvivoGen	Cat#:ant-pm-2
RLT buffer	Qiagen RNeasy Micro kit	Cat#:74004
*Ad-B3galt5*	Vector Biolabs	N/A
*Ad-Y5*	Vector Biolabs	N/A
RU-486 (Mifepristone)	Sigma-Aldrich	Cat#: M8046
TRIzol	Invitrogen	Cat#: 15596018
Qiagen RNeasy kit	Qiagen	Cat#: 74004
8M Lithium Chloride	Sigma-Aldrich	Cat#: L7026
Glycogen solution	VWR	Cat#: N632
Sodium Acetate	Sigma-Aldrich	Cat#: 127–09-3
IHC Tek Epitope retrieval solution	IHC Tek	Cat#: IW-1100
Tris base	Sigma-Aldrich	Cat#: 77–86-1
Bovine Serum Albumin	Sigma-Aldrich	Cat#: 9048–46-8
3,3′-Diaminobenzidine	Abcam	Cat#: ab64264
Mouse rIL-22.Fc	Evive Biotech	Cat#: F-652
Mouse rIL-22	Biolegend	Cat#: 576204
Human rIL-22	R & D systems	Cat#: 782-IL
DAPT	Sigma-Aldrich	Cat#: D5942
S3I-201	Sigma-Aldrich	Cat#: 501919–59-1
L-WRN conditioned medium	ATCC	Cat#: CRL-3276
Advanced DMEM/F12	ThermoFisher	Cat#: 12634028
NAC	MP Biomedicals	Cat#: 194603
FBS	R&D Systems	Cat#: S11150
Y-27632	MedChemExpress	Cat#: HY-10583
B27 formulation	ThermoFisher	17504–044 (50X)
Gastrin	AnaSpec	Cat#: AS-64149
Primocin	InvivoGen	Cat#: ant-pm-2
A 83–01	Millipore-Sigma	Cat#: SML078
SB 202190	LC Laboratories	Cat#: S-1700
PGE2	Cayman Chemical	Cat#: 14010
Nicotinamide	Millipore-Sigma	Cat#: N0636
Collagenase	Worthington	Cat#: LS004189
HEPES	Corning	Cat#: 25–060-CI
GlutaMAX	ThermoFisher	Cat#: 25050061
EGF	Peprotech	Cat#: AF-100–15
Sodium bicarbonate	Corning	Cat#: 25–035-CI
EDTA	ThermoFisher	Cat#:15575–020
Glyoxal Prefer solution	Sigma-Aldrich	Cat#:107–22-2
Rabbit anti-human Muc2	Santa Cruz Technology	Cat#: sc-15334; RRID: AB 2146667
Hoechst 33342	ThermoFischer	Cat#: 62249
Red fluorescent beads 10–45 μm diameter	Cospheric innovations in Microtechnology	N/A
Transwell Inserts	Corning	Cat#: 3460

Experimental models: Cell lines		

L-WRN cells	Dr.Thaddeus Stappenbeck Washington University in St. Louis	N/A
HT-29	ATCC	HTB-38
HEK293	ATCC	CRL-1573
Human colonic stem cells	RRID:121 CVCL_ZR41	N/A

Experimental models: Organisms/strains		

Mouse: *Villin-cre*	Jackson Laboratory	Cat#: 004586
Mouse: *Il22Ra1*^*fl/fl*^	Dr. Jay K. Kolls, Tulane University	Cat#: 031003
Mouse: *Il22*^−/−^	Dr. Jay K. Kolls, Tulane University	N/A
Mouse: *ROSA-CAG-LSL-tdTomato*	Jackson Laboratory	Cat#: 007905
Mouse: *Defa6-cre*	Dr. Richard Blumberg Harvard University	N/A
Mouse: *Lgr5-EGFP-cre*^*ERT2*^	Jackson Laboratory	Cat#: 008875
Mouse: *Myd88* ^*fl/fl*^*;Villin-cre*	Dr. Jeremy McAleer, Marshall University	N/A
Mouse: Math1-PGR-Cre	Dr. Rodney D. Newberry, Washington University	Cat#: 013594
Mouse: *C57BL/6J*	Jackson Laboratory	N/A
Mouse: B6.129(Cg)- *Gt(ROSA)26Sor*^*tm4(ACTB-tdTomato,-EGFP)Luo*^/J	Jackson Laboratory	Cat#: 007676

Oligonucleotides		

See Table S3 for qPCR primer sequences	This paper	N/A

Software and algorithms		

Prism 7	GraphPad Software	https://www.graphpad.com/scientific-software/prism/
ImageJ	NIH	https://imagej.nih.gov/ij/index.html
VIP	AS-22872	AnaSpec
cellSens Standard	Olympus	https://www.olympus-lifescience.com/en/software/cellsens/
Cell Sens Dimension v1.18 software	Olympus Corp.	N/A
NCBI GEO2R portal	NCBI	https://www.ncbi.nlm.nih.gov/geo/geo2r/
NCBI Entrez	Gene	https://www.ncbi.nlm.nih.gov/gene/
JASPAR	JASPAR 2022	https://jaspar.genereg.net/

## Highlights

- IL-22Ra1-B3GALT5 axis in intestinal epithelium protects from colitis development
- IL-22Ra1-B3GALT5 axis is involved in altering O-glycan profile of mucin
- IL-22Ra1 signaling in MATH1^+^ cells provides protection from colitis development
- IL-22Ra1 signaling in MATH1^+^ progenitor regenerates epithelium post-DSS treatment
