# IL-22 promotes mucin-type O glycosylation and MATH1^+^ cell-mediated amelioration of intestinal inflammation

**DOI:** 10.1016/j.celrep.2024.114643

**Published:** 2024-08-03

**Authors:** Ankita Singh, Michael Beaupre, Cecilia Villegas-Novoa, Kiyoshi Shiomitsu, Stephen J. Gaudino, Suzanne Tawch, Ruhee Damle, Cody Kempen, Biswa Choudhury, Jeremy P. McAleer, Brian S. Sheridan, Paula Denoya, Richard S. Blumberg, Patrick Hearing, Nancy L. Allbritton, Pawan Kumar

In the originally published version of this article, Document S1 accidentally omitted Tables S1–S3 despite declaring these items in the supplemental information index. This error occurred during the production process, where the figures were updated without including the tables in the file. The correct Document S1, including the supplementary figures and tables, is now available with the paper online. The authors and production team apologize for the omission of the supplementary tables.

