# Supplementary material for: IL-22 promotes mucin-type O-glycosylation and MATH1+ cell-mediated amelioration of intestinal inflammation
Source: Cell Rep. Author manuscript; Available in PMC 2024 Aug 16. (PMC11328608; doi:10.1016/j.celrep.2024.114206)
Supplement: 1 [file NIHMS2000703-supplement-1.pdf]

Cell Reports, Volume *43*

Supplemental information

IL-22 promotes mucin-type O-glycosylation and MATH1^{+} cell-mediated amelioration of intestinal inflammation

Ankita Singh, Michael Beaupre, Cecilia Villegas-Novoa, Kiyoshi Shiomitsu, Stephen J. Gaudino, Suzanne Tawch, Ruhee Damle, Cody Kempen, Biswa Choudhury, Jeremy P. McAleer, Brian S. Sheridan, Paula Denoya, Richard S. Blumberg, Patrick Hearing, Nancy L. Allbritton, and Pawan Kumar

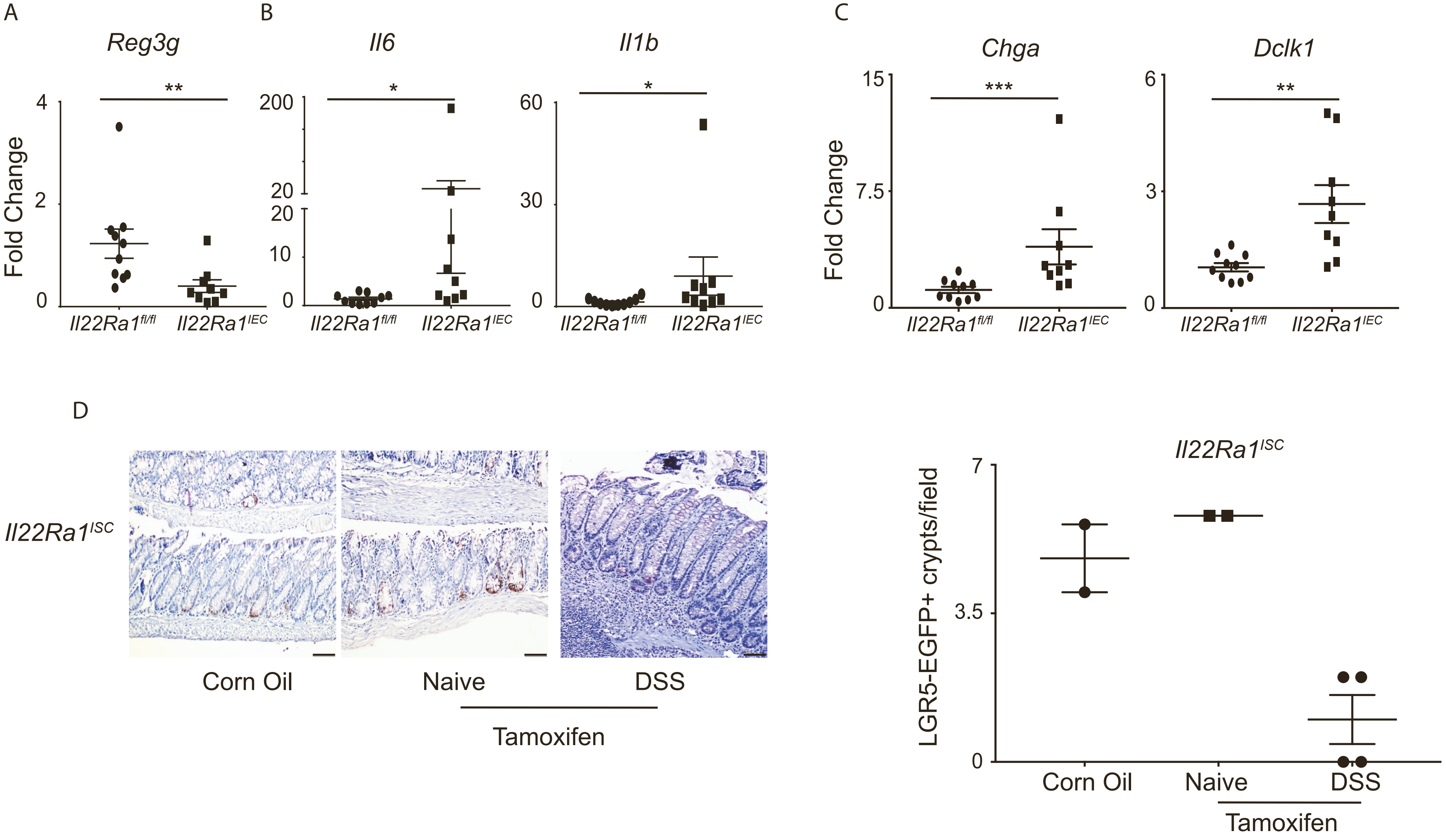

Supplementary Figure 1: Increased inflammation in DSS treated *Il22Ra1*^{IEC}mice.

RT-PCR analysis of expression of A) *Reg3g,* B) inflammatory *cytokine* genes (*Il6* and *Il1b)*, C) *Chga* (enteroendocrine cells), and *Dclk1* (tuft cells) in the distal colon tissue of DSS treated *Il22Ra1*$^{fl/fl}$ *and Il22Ra1*^{IEC} mice. D) Representative image of histochemically stained anti-LGR5^{-}GFP^{+} cells (brown) and hematoxylin stained nuclei (blue) in the colon tissue of corn oil or tamoxifen treated (naive and DSS) *Il22Ra1*^{ISC} mice (left panel, 50 μm) and their counts (right panel).

Figures A, B and C are representative of 2 independent experiments. Data are presented as Mean ± SEM in the graphs. *$P < 0.05$, **$P < 0.01$ (Mann-Whitney test, two-tailed).

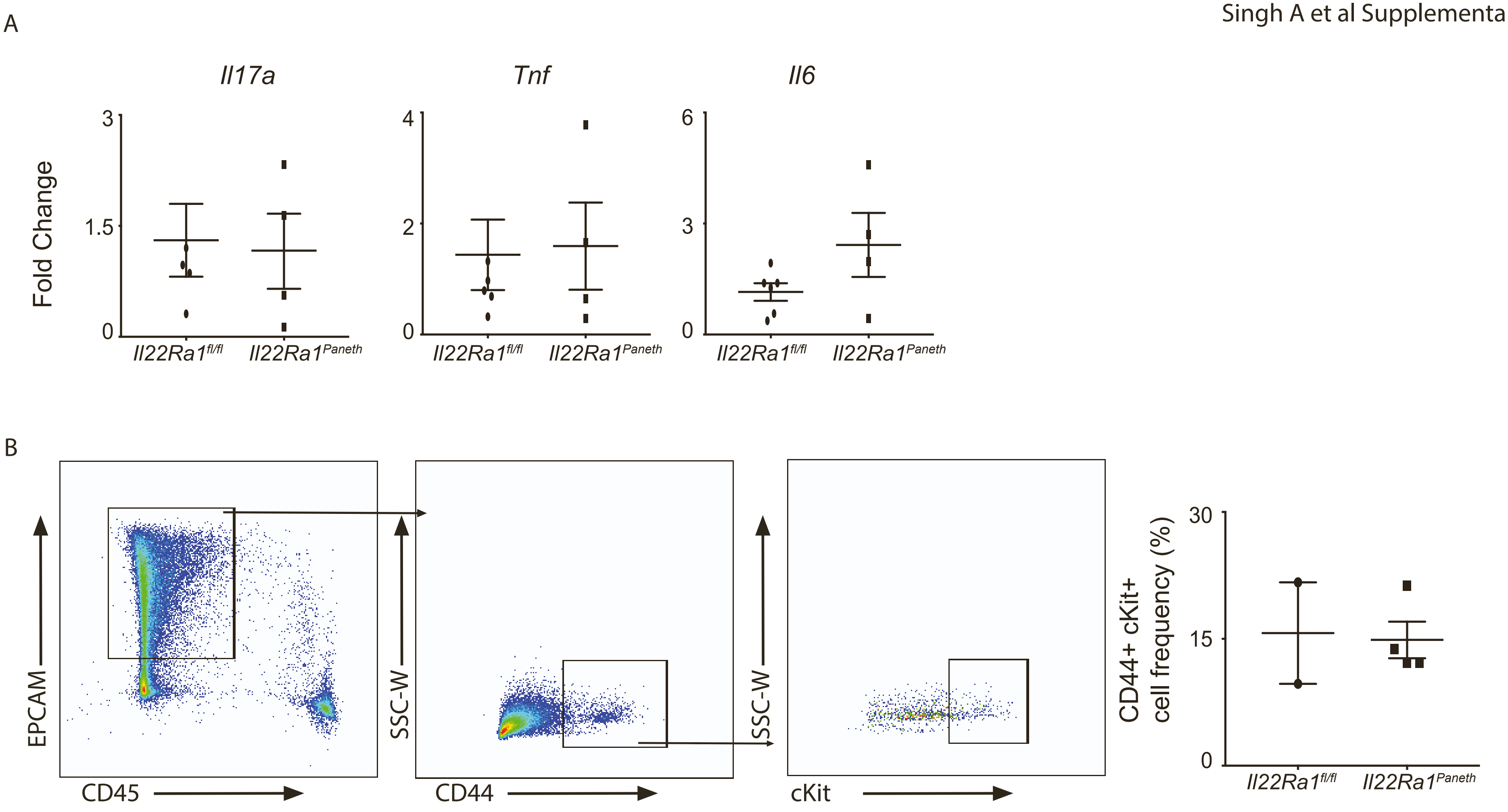

Supplementary Figure 2: Inflammatory cytokine genes expression in naive *Il22Ra1^{Paneth}* mice and Paneth-like cells in colon tissue of DSS treated *Il22Ra1^{Paneth}* mice. A) RT-PCR analysis of expression of *Il17a, Tnfa, and Il6* genes in the distal colon tissue of naive *Il22Ra1$^{fl/fl}$ and Il22Ra1^{Paneth}* mice. B) Flow data showing the gating strategy to determine the frequency of CD44^{+} and cKit^{+} cells (Paneth-like cells) in colon epithelial cells of DSS treated *Il22Ra1$^{fl/fl}$ and Il22Ra1^{Paneth}* mice.

Data are presented as Mean ± SEM in the graphs. (Mann-Whitney test, two-tailed).

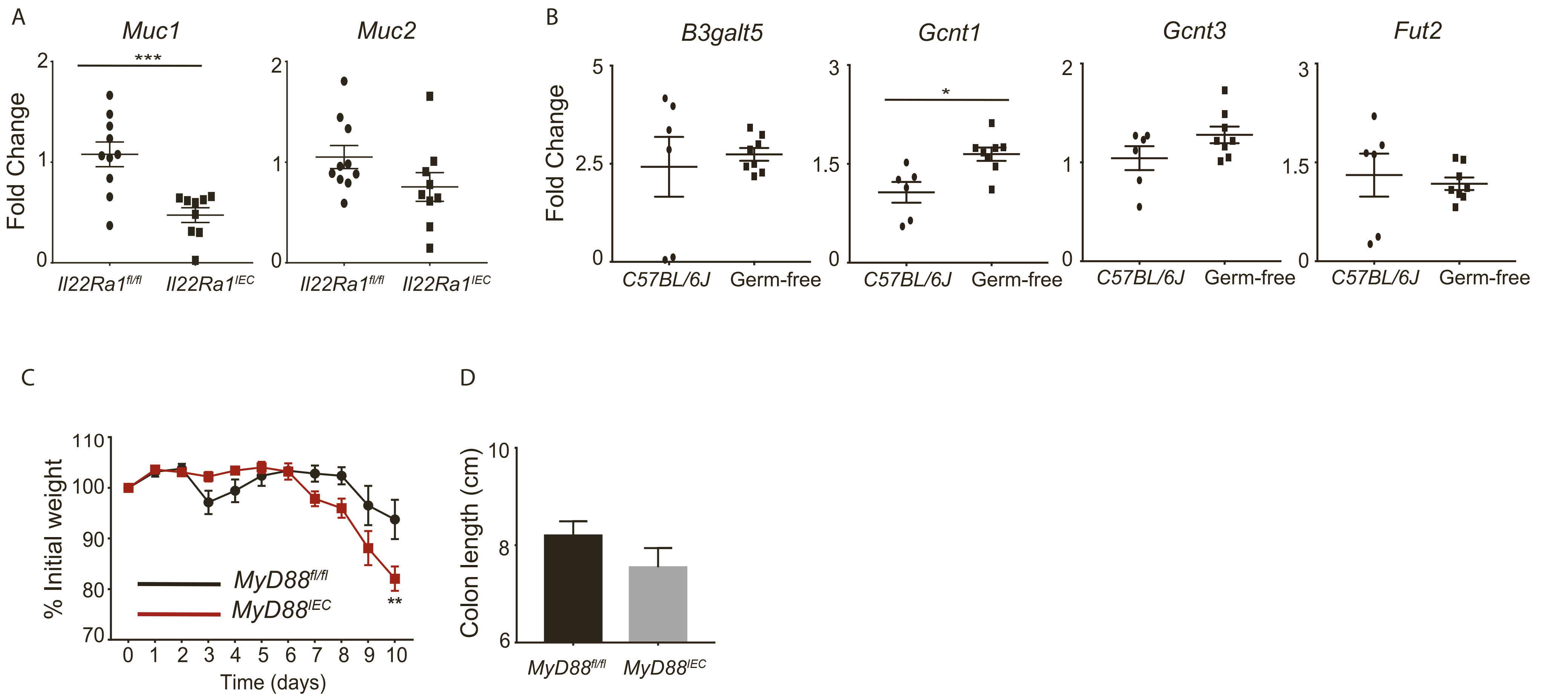

Supplementary Figure 3: Defect in mucin expression in *Il22Ra1^IEC^* mice, glycosyltransferase expression in germ-free mice and colitis development in *MyD88^IEC^* mice. A) RT-PCR analysis of expression of *Muc1 and Muc2* genes in the distal colon tissue of DSS treated *Il22Ra1*$^{fl/fl}$ and *Il22Ra1*^{IEC} mice. B) RT-PCR analysis of expression of *B3galt5, Gcnt1, Gcnt3, and Fut2* in the distal colon tissue of conventional and germ-free *C57BL/6J* mice. Analysis of C) weight loss and D) colon length of DSS treated *MyD88*$^{fl/fl}$ and *MyD88*^{IEC} mice.
Figure C and D are generated from 2 independent experiments. Data are presented as Mean ± SEM in the graphs. *$P < 0.05$, **$P < 0.01$, ***$P < 0.001$ (2-way ANOVA or Mann-Whitney test, two tailed).

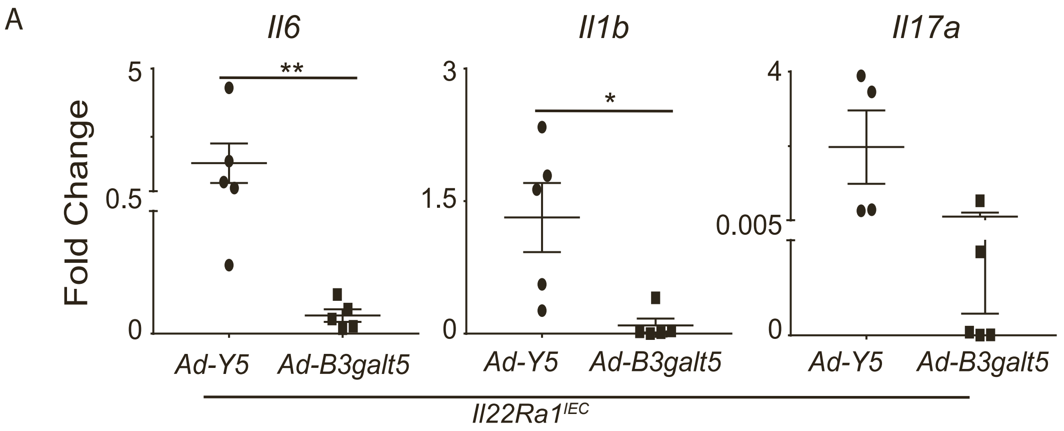

Supplementary Figure 4: Administration of *Ad-B3galt5* reduces inflammation in DSS treated *Il22Ra1^{IEC}* mice.
A) RT-PCR analysis of expression of inflammatory cytokine genes (*Il6, Il1b, Il17a*) in the distal colon tissue of *Ad-Y5/Ad-B3galt5* and DSS treated *Il22Ra1^{IEC}* mice.
Figure A is representative of 3 independent experiments. Data are presented as Mean ± SEM in the graphs. *$P < 0.05$, **$P < 0.01$ (Mann-Whitney test, two tailed).

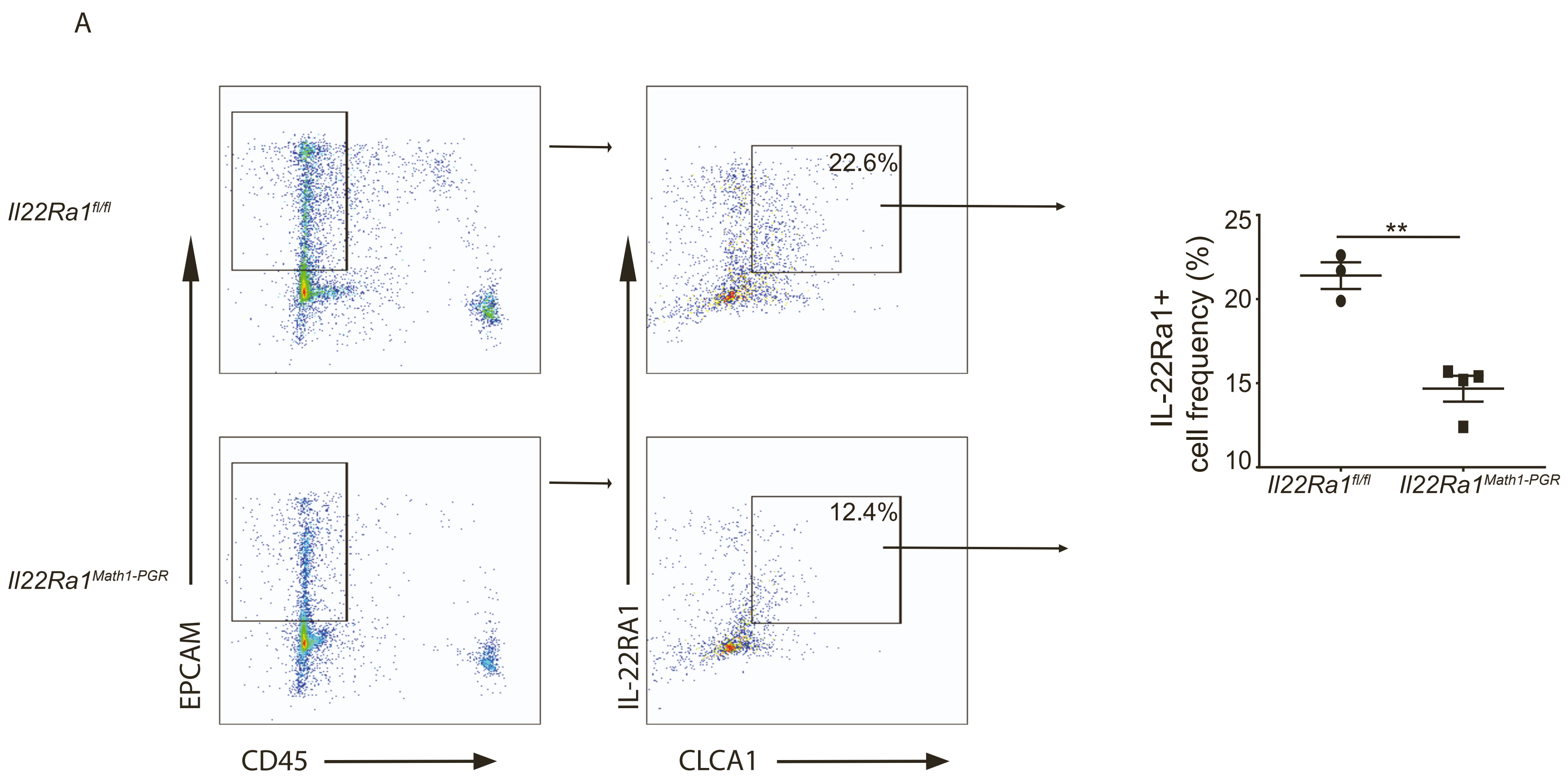

Supplementary Figure 5: Validation of IL-22Ra1 expression on goblet cells in RU-486 treated *Il22Ra1*$^{Math1\text{-}PGR}$ mice. A) Flow representative plots showing the gating strategy for IL-22Ra1 expression in CLCA1^{+} (goblet cells) colon epithelial cells in RU-486 injected *I22Ra1*$^{fl/fl}$ and *Il22Ra1*$^{Math1\text{-}PGR}$ mice.
Figure A is representative of 2 independent experiments. Data is presented as Mean ± SEM in the graph. ***P* < 0.01 (*Student t-test*).

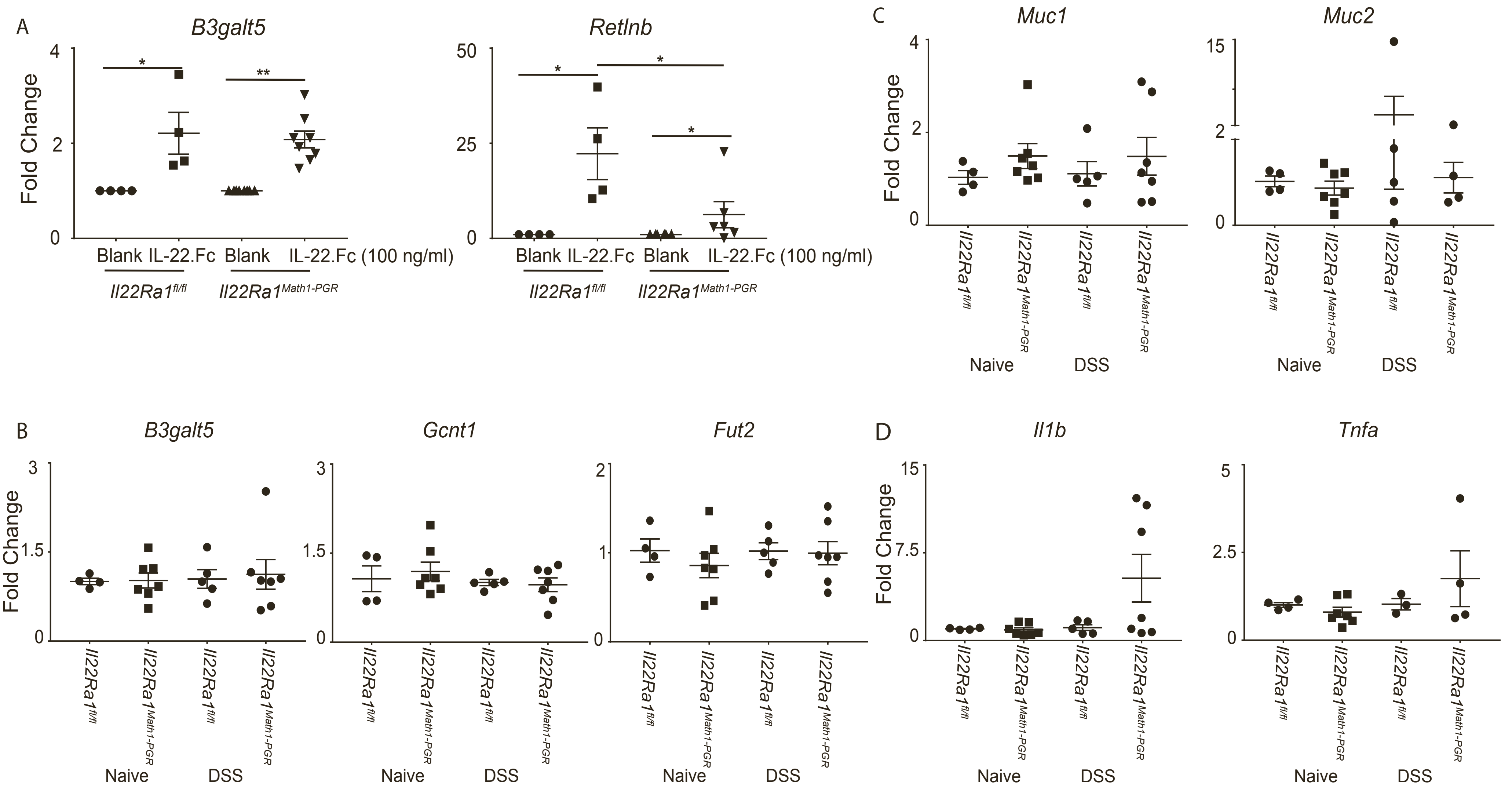

Supplementary Figure 6: IL-22Ra1 signaling induces *B3galt5* expression on both MATH1^{+} and MATH1^{-} cell populations.
A) RT-PCR analysis of expression of *B3galt5* and *Retlnb* genes in colon organoids of *Il22Ra1$^{fl/fl}$ and Il22Ra1$^{Math1\text{-}PGR}$* mice after RU-486 treatment and with/without IL-22.Fc (100 ng/ml). RT-PCR analysis of expression of B) glycosyltransferase, C) mucin and D) inflammatory cytokine genes in the distal colon tissue of naïve (after 5 days of RU-486 injection) and DSS treated *Il22Ra1$^{fl/fl}$ and Il22Ra1$^{Math1\text{-}PGR}$* mice. Figure A and B are representative of at least 2 independent experiments. Data are presented as Mean ± SEM in the graphs. *$P < 0.05$, **$P < 0.01$ (Mann-Whitney test, two tailed).

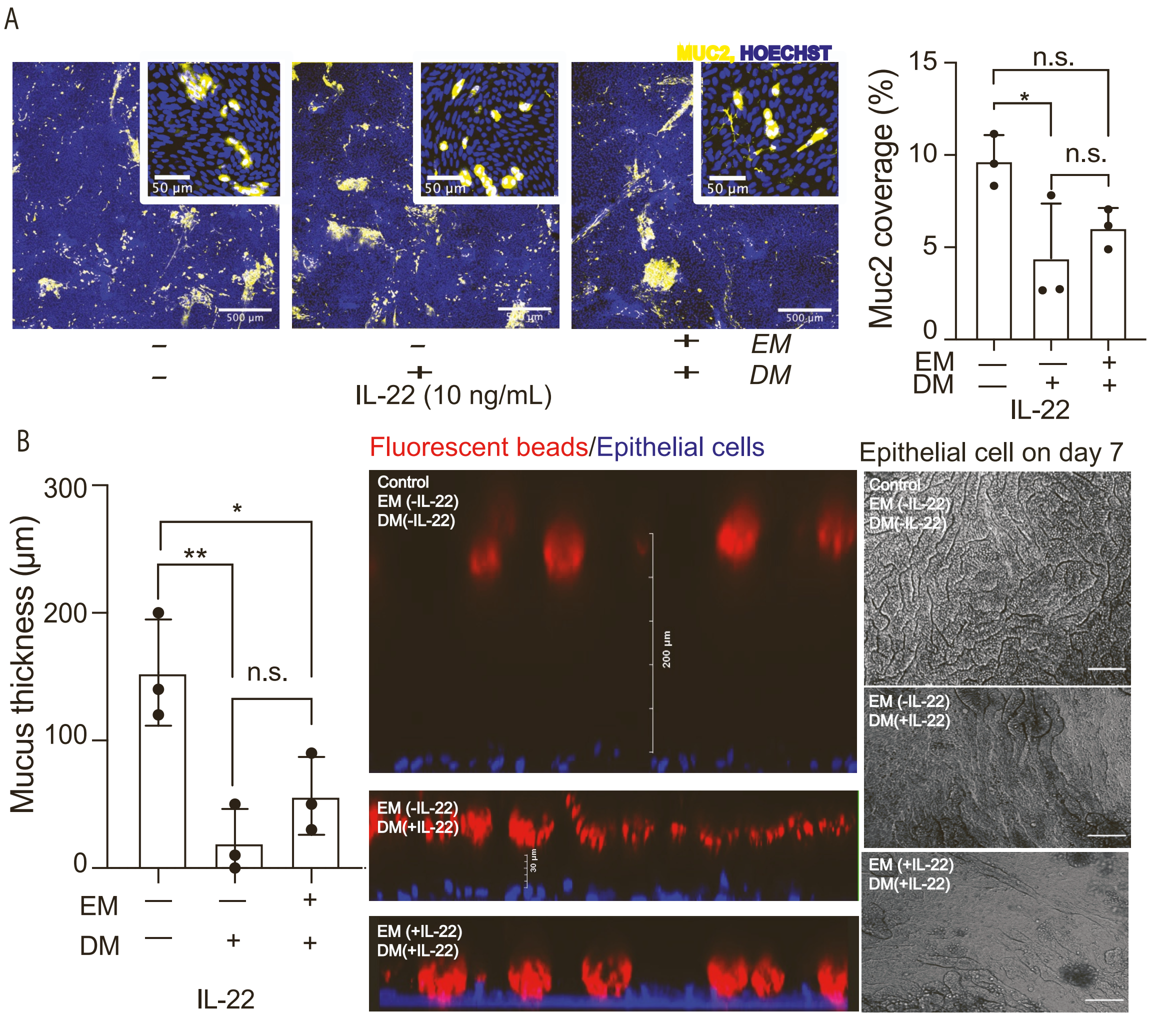

Supplementary Figure 7: Changes in mucus layer thickness by IL-22Ra1 signaling in Human intestinal epithelial cells.
A) Representative image of the monolayer of human intestinal cells immunofluorescence stained with anti-MUC2 antibody (yellow) and Hoechst 33342 with/without IL-22 stimulation (10 ng/ml) during expansion medium (EM) or differentiation medium (DM). MUC2 coverage area was determined in response to IL-22 stimulation (right panel) and normalized to an area positive for Hoechst 33342. B) Analysis of mucus layer thickness by measuring the distance between dry red fluorescent beads on top of mucus and blue Hoechst-stained living epithelial cell nuclei (30 µm).
In figure A and B each conditions were performed in triplicate. Data are presented as Mean ± SEM in the graphs. *$P < 0.05$, **$P < 0.01$ (Mann-Whitney test, two tailed).

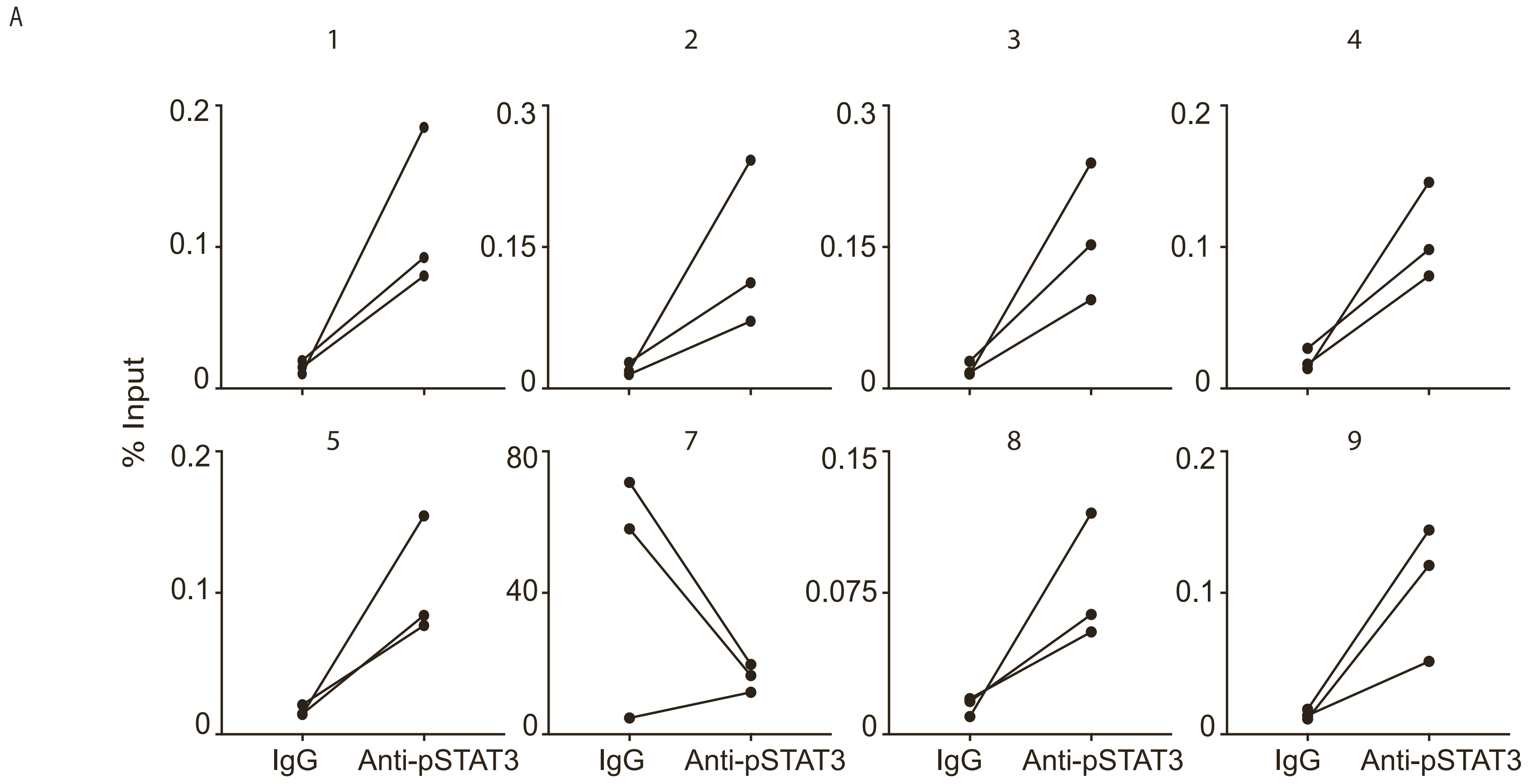

Supplementary Figure 8: Putative STAT3 binding sites in the promoter region of *B3GALT5* gene.
A) HT-29 cells were cultured with human rIL-22 (50 ng/ml) for 24 hr. Cells were harvested and processed for ChIP assay with anti-pSTAT3 antibody. The pSTAT3 binding DNA was utilized to analyze 9 (8 sites primer data) putative binding sites in the promoter region of *B3GALT5* gene by qPCR.
Data are presented as Mean ± SEM in the graphs. (Paired Student t-test).

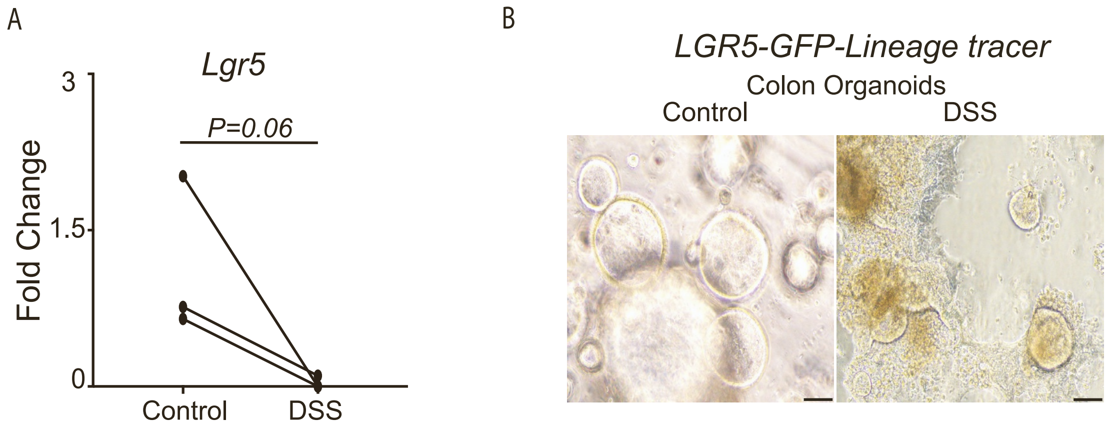

Supplementary Figure 9: Depletion of LGR5⁺ ISC on DSS treatment of colon organoids of *LGR5-GFP*-Lineage tracer mice.
A) RT-PCR analysis of *Lgr5* expression and B) Microscopic bright field images of *Lgr5-GFP*-Lineage tracer mice colon organoids after 24 hr of treatment with/wihtout 0.1% of DSS (3 hr, 50 μm).
Figure A and B are representative of 1 independent experiment with 3 mice in each group. Data is presented as Mean ± SEM in the graph. (*Student t-test*).
