# Supplementary material for: IL-22 promotes mucin-type O-glycosylation and MATH1+ cell-mediated amelioration of intestinal inflammation
Source: Cell Rep. Author manuscript; Available in PMC 2024 Aug 16. (PMC11328608; doi:10.1016/j.celrep.2024.114206)
Supplement: Supplementary Tables [file NIHMS2000703-supplement-Supplementary_Tables.pdf]

Sample	Disease tissue	Age	Gender	Diagnosis
Patient 1	A6	58	Male	Ulcerative colitis (UC)
Patient 2	A2	37	Male	UC with acute inflammation of mucosa
Patient 3	B4	52	Female	UC with extensive pseudopolyps in transverse colon
Patient 4	A17	52	Male	Severe active UC with cryptitis and crypt abscess
Patient 5	A10	41	Male	UC with severe pancolitis
	Normal tissue			
Control 1	C2	35	Male	Diverticulitis with abscess and acute/chronic serositis
Control 2	C2	60	Male	Diverticulitis with abscess formation and fibrosis
Control 3	B1	45	Male	Diverticulitis with abscess formation and serositis
Control 4	B1	40	Male	Diverticulitis with abscess formation
Control 5	D4	52	Female	Diverticulitis with fistula and abscess formation

Supplementary Table 1. Clinical details of patients from which colon tissue samples were included in the study for Tn antigen staining.

		EM media	DM-SUB (Submerged)	DM-ALI (Air-liquid interface)
	Final Concentrations	For expansion phase	For differentiation	For differentiation
L-WRN conditioned Media	50% (0% for DM only)	250 mL		
Advanced DMEM/F12	50% (100% for DM only)	250 mL	500 mL	500 mL
Glutamax	1x	2.5ml	5 ml	5 mL
HEPES	10 mmol/L	2.5 mL	5 mL	5 mL
Primocin	50 ug/ml	500ul	500ul	500ul
NAC	1.25 mmol/L	500ul	500ul	
EGF	50 ng/ml	100ul	100ul	100ul
Nicotinamide	10 mmol/L	5 mL		
B27	1x	10 mL		
Gastrin	10 nmol/L	12.5ul		
PGE2	10 nmol/L	5ul		
A83-01	500 nmol/L		50ul	50ul
SB202190	3 umol/L	50ul		
Y-27632	10 umol/L	500 uL		
FBS (Heat inactivated)	10%		50 mL	50 mL
VIP	330 ng/mL			40 uL into 40 mL of DM-ALI medium

Supplementary Table 2. Formulation of culture medium for human colonic epithelial cells.

Oligonucleotides	Source	Identifier
Hprt (Mm00446968_m1)	Applied Biosystems	N/A
Lgr5 (Mm00438890_m1)	Applied Biosystems	N/A
Il22Ra1 (Mm.PT.58.42129001)	Applied Biosystems	N/A
Reg3γ (Mm.PT.58.1275735)	Applied Biosystems	N/A
Il17a (Mm00439618_m1)	Applied Biosystems	N/A
Ki67 (Mm_Mki67_1_SG)	Applied Biosystems	N/A
Il22 (Mm00444241-m1)	Applied Biosystems	N/A
Atoh1 (Mm00476035)	Applied Biosystems	N/A
Dclk1 (Mm00444950_m1)	Applied Biosystems	N/A
Chga (Mm0051431_m1)	Applied Biosystems	N/A
Tnfa (Mm.PT.58.12575861)	IDT	N/A
Muc2 (Mm.PT.58.53535475.g)	IDT	N/A
Lyz1 (Mm.PT.58.31099880)	IDT	N/A
Mouse Gapdh<br>FP-5’-TGACCTCAACTACATGGTCTACA-3’	IDT	N/A
Mouse Gapdh<br>RP-5’-CTTCCCATTCTGGGCCTTG-3’	IDT	N/A
Mouse Muc1<br>FP-5’- TCCTTGCCCTGGCAGTGTGC-3’	IDT	N/A
Mouse Muc1<br>RP-5’- CCGCCAAAGCTGCCGCAAGT -3’	IDT	N/A
Mouse B3galt5<br>FP-5’- TCACTCACCGGCTGCTCTTT -3’	IDT	N/A
Mouse B3galt5<br>RP-5’- TGAGCCATCTTTGCCGAGTA -3’	IDT	N/A
Mouse Fut2<br>FP-5’- ACAGCCAGAAGAGCCATGGC -3’	IDT	N/A
Mouse Fut2	IDT	N/A

RP-5'- TAACACCGGGAGACTGATCC -3'		
Mouse Gcnt1<br><br>FP-5'- GCATCGCATCCTGCTTTGATA -3'	IDT	N/A
Mouse Gcnt1<br><br>RP-5'- GGTCTGCCTTAACCCGACTC -3'	IDT	N/A
Mouse Gcnt3<br><br>FP-5'- AGAGTTCCATCAACTGCTCAGG -3'	IDT	N/A
Mouse Gcnt3<br><br>RP-5'- CATCCTAAGGTAGTCGGCCTC -3'	IDT	N/A
Mouse Retlnb<br><br>FP-5'- CCATTTCCTGACCTTTCTGG -3'	IDT	N/A
Mouse Retlnb<br><br>RP-5'- AGCACATCCAGTGACAACCA -3'	IDT	N/A
Mouse Il1b<br><br>FP-5'-GCAACTGTTCCTGAACTCAACT-3'	IDT	N/A
Mouse Il1b<br><br>RP-5'-ATCTTTTGGGGTCCGTCAACT-3'	IDT	N/A
Mouse Il6<br><br>FP-5'-TCCAATGCTCTCCTAACAGATAAG -3'	IDT	N/A
Mouse Il6<br><br>RP-5'- CAAGATGAATTGGATGGTCTTG -3'	IDT	N/A
Human HPRT<br><br>FP-5'- GAAAAGGACCCCACGAAGTGT -3'	IDT	N/A
Human HPRT<br><br>RP-5'- AGTCAAGGGCATATCCTACAACA -3'	IDT	N/A
Human B3GALT5<br><br>FP-5'- CCGGCTACGTGTTTTCTGG -3'	IDT	N/A
Human B3GALT5<br><br>RP-5'- GAAGAGGCATACGGAGAAGCG -3'	IDT	N/A

Human FUT2 FP-5’- TCCCCTGGCAGAACTACCA -3’,	IDT	N/A
Human FUT2 RP-5’- GGTGAAGCGGACGTACTCC -3’	IDT	N/A
ChIP assay primers:		
1A FP-5’-CATAACTTTAAATGCACTGGGAGAT-3’	IDT	N/A
1A RP-5’-CCATTACAGACTACTGCAATAAAGC-3’	IDT	N/A
2A FP-5’-TTGCTTTATTGCAGTAGTCTGT-3’	IDT	N/A
2A RP-5’-ATGGGTCTCCAACTTGCT-3’	IDT	N/A
3A FP-5’-CTGTGTTTGAAGGCCTGAGA-3’	IDT	N/A
3A RP-5’-TGCCTTCGGACTTGAACTG-3’	IDT	N/A
4A FP-5’-TGATGCTCAGCCCACCT-3’	IDT	N/A
4A RP-5’-GACCCTCTAAGTAATTAAGAAGTCACA-3’	IDT	N/A
5A FP-5’-GTGAGTCGTCCTGCAGCTTAG-3’	IDT	N/A
5A RP-5’-CTGGCTGGGCCTTTGGA-3’	IDT	N/A
6A FP-5’-GGTCTGAAGCTTATACAATTGGG-3’	IDT	N/A
6A RP-5’-CCACGAGACTAGTGAAGCTAAG-3’	IDT	N/A
7A FP-5’-TGCTGTTCTGTTCTCCCAAG-3’	IDT	N/A
7A RP-5’-GAGTGCTAGAGTCTGCTTTCAG-3’	IDT	N/A
8A FP-5’-CTTCACTCCAGATCTCTCCTTTC-3’	IDT	N/A
8A RP-5’-AAGTCCACCTCTCTAAGGACT-3’	IDT	N/A
9A FP-5’-GGTATTTCCCAACCTCCGTAAT-3’	IDT	N/A
9A RP-5’-GGTAAGTCGGTAGGTAGACAGA-3’	IDT	N/A

Supplementary Table 3: Sequences of qPCR primers.
